# Cardiovascular disease detection from cardiac arrhythmia ECG signals using artificial intelligence models with hyperparameters tuning methodologies

**DOI:** 10.1016/j.heliyon.2024.e36751

**Published:** 2024-08-22

**Authors:** Gowri Shankar Manivannan, Harikumar Rajaguru, Rajanna S, Satish V. Talawar

**Affiliations:** a Malnad College of Engineering, Hassan, Karnataka, India; b Bannari Amman Institute of Technology, Sathyamangalam, Tamilnadu, India

**Keywords:** Adam, Electrocardiogram, Grid search optimization, Hyperparameters, Cardio vascular diseases

## Abstract

Cardiovascular disease (CVD) is connected with irregular cardiac electrical activity, which can be seen in ECG alterations. Due to its convenience and non-invasive aspect, the ECG is routinely exploited to identify different arrhythmias and automatic ECG recognition is needed immediately. In this paper, enhancement for the detection of CVDs such as Ventricular Tachycardia (VT), Premature Ventricular Contraction (PVC) and ST Change (ST) arrhythmia using different dimensionality reduction techniques and multiple classifiers are presented. Three-dimensionality reduction methods, such as Local Linear Embedding (LLE), Diffusion Maps (DM), and Laplacian Eigen (LE), are employed. The dimensionally reduced ECG samples are further feature selected with Cuckoo Search (CS) and Harmonic Search Optimization (HSO) algorithms. A publicly available MIT-BIH (Physionet) - VT database, PVC database, ST Change database and NSR database were used in this work. The cardiac vascular disturbances are classified by using seven classifiers such as Gaussian Mixture Model (GMM), Expectation Maximization (EM), Non-linear Regression (NLR), Logistic Regression (LR), Bayesian Linear Discriminant Analysis (BDLC), Detrended Fluctuation Analysis (Detrended FA), and Firefly. For different classes, the average overall accuracy of the classification techniques is 55.65 % when without CS and HSO feature selection, 64.36 % when CS feature selection is used, and 75.39 % when HSO feature selection is used. Also, to improve the performance of classifiers, the hyperparameters of four classifiers (GMM, EM, BDLC and Firefly) are tuned with the Adam and Grid Search Optimization (GSO) approaches. The average accuracy of classification for the CS feature-based classifiers that used GSO and Adam hyperparameter tuning was 79.92 % and 85.78 %, respectively. The average accuracy of classification for the HSO feature-based classifiers that used GSO and Adam hyperparameter tuning was 86.87 % and 93.77 %, respectively. The performance of the classifier is analyzed based on the accuracy parameter for both with and without feature selection methods and with hyperparameter tuning techniques. In the case of ST vs. NSR, a higher accuracy of 98.92 % is achieved for the LLE dimensionality reduction with HSO feature selection for the GMM classifier with Adam's hyperparameter tuning approach. The GMM classifier with the Adam hyperparameter tuning approach with 98.92 % accuracy in detecting ST vs. NSR cardiac disease is outperforming all other classifiers and methodologies.

## 1 Introduction

A rapid, abnormal cardiac rhythm is known as VT. VT is characterized as three or more consecutive heartbeats with more than 100 heartbeats per minute. If VT lasts for more than a few seconds, it can be fatal. Due to fast heart beating, cardiac circulation loses the synchronization that beats to ventricular fibrillation [1]. Symptoms of VT include cardiac arrest, chest pain, and breathing shortness. PVC is one of the ventricular arrhythmia. It is an irregular cardiac rhythm. Symptoms of PVC include fluttering and skipped beats. ST Segment represents the duration between ventricular depolarization and ventricular repolarization. Myocardial infarction is the most common cause of ST change abnormalities. These ST Change abnormalities are also known as depression or elevation [1]. In the last three decades, the scientific community has introduced a punch of algorithms to detect cardiac arrhythmias such as VT, PVC and ST from ECG signals.

The electrocardiogram (ECG) is an investigative instrument that monitors and records the electrical activities of the human heart [2]. ECG is useful for identifying the source of chest pain and for detecting irregular heart rhythms or cardiac irregularities. Usually, healthy hearts undergo cardiac ECG. Any heart rhythm irregularity can alter the shape of the ECG Signal [2]. It is based on a standard 12 lead system, which tests the electrical potential of the 10 electrodes placed on various parts of the body surface, six in the chest and four in the limbs. An early diagnosis is necessary in order to provide efficient care of arrhythmias [3]. There are three wave in each cardiac cycle, including the P wave, QRS complex and T wave [4]. ECG arrhythmia detection is an important part of the identification of different cardiac illnesses. Effective and precise ECG arrhythmia diagnosis allows doctors to diagnose various heart disorders. The detection of arrhythmia using ECG is very difficult. This is due to the variability in the typical ECG waveform of each individual, the dissimilar signs for one disease happening different electrocardiogram waveform patients, two dissimilar illnesses ought to roughly similar effects happening different electrocardiogram waveform patients, inconsistency of ECG Characteristics and complete absence of effective detection algorithm for beat of ECG classification [5].

Several detection techniques for cardiovascular diseases have been mostly presented in recent years. Most of these methods are made up of four steps: preprocessing (de-noising), dimensionality reduction, feature selection, and identifying different cardiac arrhythmias. Discriminant analysis was used to extract the ventricular fibrillation with the help of MIT-BIH ECG signals by Irena et al. [6], and the best detection output values were achieved with an average sensitivity of 94.1 % and an average specificity of 93.8 %. A Support Vector Machine (SVM) with 14 metrics was proposed by Qiao et al. [7] for the detection of ventricular fibrillation and ventricular tachycardia and found that the average detection accuracy was 95 % only. A Signal Comparison Algorithm (SCA) approach to the detection of VT based on publicly available annotated datasets was done by Tratning et al. and found that the average detection accuracy was 96.2 %, sensitivity of 71.2 % and Specificity of 98.5 % [8]. Shweta et al. used a hybrid of Particle Swarm Optimization (PSO) and Feed Forward Neural Network (FFNN) classifiers for ECG beat detection and found an overall detection accuracy of 97 % [2]. The SVM, Adaboost, ANN and Naïve Bayes classifiers for ECG signals classification were done by Celin et al. and the naïve Bayers result achieved a high accuracy of 99.7 % compared with the SVM, Adaboost and ANN Classifiers [9]. A Genetic Algorithm and Kernel Extreme Learning Machine (KELM) were used to detect the arrhythmias with the help of ECG signals by Dikera et al. [10], and the best detection output values were achieved an accuracy of 95 %, sensitivity of 100 % and Specificity of 80 %. For the automated diagnosis of heart diseases using MIT-BIH ECG Signals, fast compression residual convolutional neural networks (FCResNN) were proposed by Jing et al. and they achieved an accuracy of 98.79 % [11]. Discrete Wavelet Transform (DWT) and Principal Component Analysis (PCA) were used to extract the ECG signal features and classify five classes of cardiac arrhythmias using the SVM-RBF classifier with 10-fold cross-validation by Martis et al. [12]. The average classification accuracy was reached 96.92 %. Fuzzy Hybrid neural network with Higher Order Spectra features (HOS) classifiers for seven classes of ECG beat Recognition was done by Trans et al. [13] and found that the overall recognition accuracy was 96.06 %. Discrete Wavelet Transform (DWT) with Neural Network classifier was used to classify the four classes of cardiac abnormalities through 10-fold cross-validation by Sukanta et al. [14] and found that the average classification accuracy was 96.67 %.

Hjorth Descriptor with Artificial Neural Network (ANN) and K-Nearest Neighbours (KNN) classifiers for three classes of ECG signal classification were done by Rizal et al. [15], and the best detection output value achieved an average accuracy of 93.3 % for 10 fold cross-validation. A Bispectrum PCA with SVM-RBF was suggested by Martis et al. [16] for the detection of five classes of ECG abnormalities and found that the average detection accuracy was 93.48 % using 10-fold cross-validation. The higher order cumulative with PCA and Neural Network (NN) was used to classify the five classes of cardiac abnormalities Martis et al. [17], and the average overall classification accuracy achieved 94.52 % using 10-fold cross-validation. Nazmy et al. [18] proposed an ICA, Power Spectrum with FFNN, FIS and ANFIS classifiers to classify six types of ECG abnormalities. The best detection output values were achieved with an accuracy of 97.1 % (ANFIS). Autoregressive modelling with the GLM algorithm was used to classify the six classes of ECG Signals by Dingfei et al. [19], and the average overall classification accuracy achieved 93.2 %.

A classifier called particle swarm optimization with chi-square distance for arrhythmia classification was proposed by Dhiah et al. [20], achieving the best detection accuracy of 98 %. The Pan-Tompkins algorithm, as well as features based on time-domain HRV, were utilized by Masud et al. [21] to extract short-term atrial fibrillation signal characteristics, which were classified using an Adaboost classifier involving 5-fold cross-validation, this resulted in average classification accuracy levels reaching 91 %. Shikha et al. [22] applied the three-dimensional discrete wavelet transform (3D DWT) method on ECG abnormalities while employing a support vector machine (SVM) as a classifier, achieving an average classification accuracy of 99 %. FIR filtering, together with the KNN classifier, was proposed for classifying the ECG family by Alba et al. [23], attaining the highest level of detection accuracy equal to 89 %. A deep neural network model with residual blocks was presented by Mohamed et al. [24] for the detection of six classes of ECG abnormalities, and the average detection accuracy found was 99.51 %. Manas et al. [25] proposed a scalar invariant transform with deep neural network classifiers with 5-fold cross-validation to classify three ECG abnormalities that had recorded high detection accuracies, reaching 99.78 %.

Due to their usage of the highest dimensionality, irrelevant characteristics, missing data, and redundancy, the aforementioned machine learning-based algorithms have demonstrated a considerable increase in their ability to diagnose cardiovascular diseases accurately. Consequently, a machine learning-based system capable of effectively detecting individuals with cardiac diseases must be developed. Also, none of the abovementioned research that has been done so far has used hyperparameter modification to improve the accuracy of cardiovascular disease diagnosis. Tuning the hyperparameters of a machine learning classifier is a more effective method for improving its performance. Data analysts configure hyperparameters before the learning procedure, which is independent. After trying out a few different hyperparameter values, the results are compared so that the best solution can be found. The method of tuning hyperparameters is mostly based on experimental outcomes rather than theoretical results [26]. People expect high-quality treatment and services in the medical field [27]. Therefore, the main goal of the study is to use the GSO and Adam approaches to make the GMM, EM, BDLC, and Firefly classification algorithms more effective. Following the execution of the GSO and Adam approaches, it will be possible to choose the optimum values for the classification algorithm criteria. By utilizing these optimized hyperparameters, the method for identifying cardiovascular disease can be made to work better. The following are summaries of the study contributions.

i) Various CVDs-based ECG signals are reduced in dimensions via LLE, DM, and LE.
ii) The number of dimensions of different CVDs-based ECG signals is further reduced through the feature selection process of CS and HSO algorithms.
iii) Then, the dimensionally reduced values and the CS and HSO feature-selection values are given to the different classifiers like GMM, EM, NLR, LR, BDLC, Detrended FA and Firefly to detect ventricular arrhythmias from ECG signals.
iv) Hyperparameter tuning strategies are also used for the GMM, EM, BDLC, and Firefly classification algorithms. In this study, the GSO and Adam approaches are used to determine the optimized hyperparameter results for each classification algorithm.
v) Finally, the classifier outcome is examined and validated with and without feature selection, as well as with hyperparameter tuning. Here, OA, F1 score, GDR, MCC, and error rate are the performance metrics of the several classifiers.

The organization of work is as follows. In section 2, materials and methods are described. In section 3, the use of the dimensionality reduction technique is discussed, section 4 deals with feature selection optimization methods, and section 5 explains how to use classifiers for classification. In contrast, sections 6, 7, 8, 9 provide training and testing, hyperparameters tuning methodologies, results and discussion, and conclusion.

## 2 Materials and methods

**Fig. 1:**
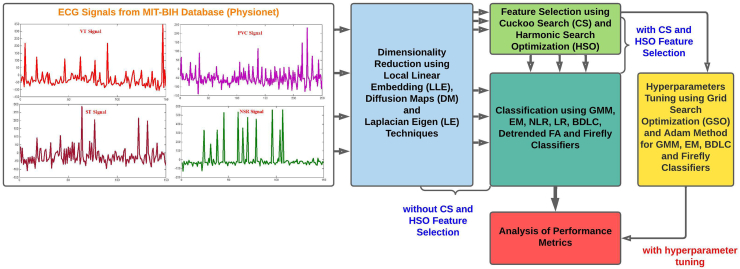
Overall methodology of the work.

The ECG raw signal database is drawn from MIT-BIH (Physionet) different cardiac class databases. In this work, four different databases are utilized. The four different databases are the VT database, PVC database, ST Change database and NSR database. 360 Hz samples are digitized for recording per channel per second with an11 bit resolution of 10 mV [28]. In this work, we utilized 74 subjects with 148 recordings. The MIT-BIH Ventricular Tachycardia (VT) database consists of 12 subjects, and each has been with two records (ML I, V1) for a total of 24 recordings. The MIT-BIH premature ventricular Contraction (PVC) database consists of 16 subjects, and each has two records (ML I, V1, V4 or V5) for a total of 32 recordings. The MIT-BIH ST Change (ST) database consists of 28 subjects, and each has been with two records (ECG1, ECG2), for a total of 56 recordings. The MIT-BIH Normal Sinus Rhythm (NSR) database consists of 18 subjects, and each has two records (ECG1, ECG2), for a total of 36 recordings. Therefore, these subjects and recordings have enough Normal VT, PVC and ST Arrhythmia beats for the work. The sampling frequency of the given VT, PVC and ST ECG signal is 360 HZ, and the Normal ECG signal is 128 HZ. The details of the MIT-BIH database of our work are shown in Table 1. The dimensionality of ECG data is quite large and occupies a larger memory space. The fundamental goal of dimensionality reduction techniques is to convert a high-dimensional data space into a low-dimensional data space. The dimensionality of the reduced form should equal that of the original data's inherent dimensionality. Dimensionality reductions are significant in many applications since they reduce undesirable characteristics and the curse of dimensionality [29]. For this work, four distinct diseases were used to construct classification issues such as VT vs. NSR, PVC vs. NSR, and ST vs. NSR. The overall methodology for automated detection of different cardiac arrhythmias is shown in Fig. 1.

**Table 1:** Details of MIT-BIH database.

Database (Classes)	Subjects (Total Number of Patients)	Total Recordings	Total Number of Epochs for Recordings	Sampling Frequency (Hz)	Sampling Interval (Sec)
VT	12	24	43333	360	0.0028
PVC	16	32	57778	360	0.0028
ST	28	56	84000	360	0.0028
NSR	18	36	141750	128	0.0078

In this work, for the purpose of dimensionality reduction and feature selection, the cardiac arrhythmia samples are is divided into epochs. VT signal is divided into 43333 epochs, each with 360 samples; PVC signal consists of 57778 epochs each having 360 samples, ST Change signals divided into 84000 epochs each with 360 samples and NSR signal consists of 141750epochs each having 128 samples. Local Linear Embedding (LLE), Diffusion Map (DM) and Laplacian Eigen (LE) are used to reduce the dimension of the ECG data. After the dimensionality reduction VT signal consists of 2167 epochs, PVC signal consists of 2889 epochs, ST change signal consists of 4200 epochs and NSR signal consists of 7088 epochs. Then feature selection is initiated using Cuckoo Search (CS) technique. After CS feature selection, VT signal consists of 333 epochs, PVC signal consists of 444 epochs, ST change signal consists of 778 epochs and NSR signal consists of 1406 epochs. The dimensionality-reduced ECG samples with and without CS feature selection are given as input to the non-linear classifiers to detect possible ventricular arrhythmias. The following section explains three different dimensionality techniques.

## 3 Dimensionality reduction techniques

### 3.1 Local Linear Embedding (LLE)

**Fig. 2:**
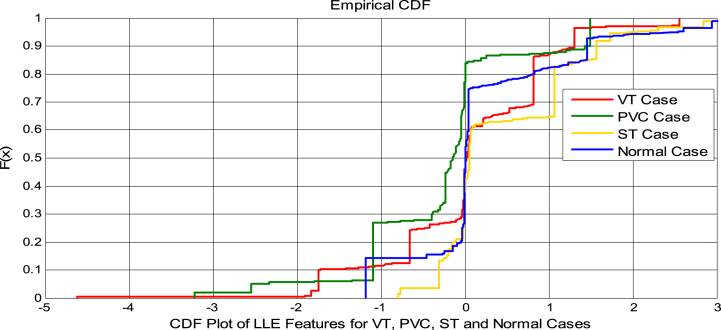
Cdf plot evaluation of LLE features for VT, ST, PVC and NSR

It is one local non-linear technique of dimensionality reduction. LLE preserves the local properties of data and the global layout of the data. LLE is a less sensitive one. The data manifold's local properties are built through the data points also as a combination of linear for the k nearest [30]. In the LLE method, there are three essential steps. A neighbourhood is constructed for each point of data, and the weights of estimating data in such a linear way. So in this neighbourhood are calculated. Finally, the weights that aid in the most accurate reconstruction of low-dimensional coordinates have been discovered. To a dxj data matrix W, the inputs to the LLE algorithms are provided [31]. Fig. 2 represents the Cumulative Distribution Function (CDF) plot evaluation of LLE features for VT, PVC, ST and Normal cases. As shown in Fig. 2 that the features among the all the classes are overlapped, non-Gaussian and nonlinear. Hence, it is advised to use good classifiers for better results.

i. For ${\vec{z}}_{i}$ , the j nearest neighbours was discovered.
ii. ${\vec{z}}_{i}$ from its neighbours

(1) $$S\left(j\right)={\sum_{k=1}^{r}\left\|{\vec{z}}_{i}-\;\sum_{l\neq k}{\vec{x}}_{ij}{\vec{z}}_{j}\right\|}^{2}$$

iii. As a result, minimizing the cost function is equivalent to finding the dimensional data representation Q.

(2) $$\varphi \left(Q\right)={\sum_{k=1}^{n}\left\|{\vec{Q}}_{i}-\;\sum_{l\neq k}{\vec{x}}_{ij}{\vec{Q}}_{j}\right\|}^{2}$$

Where $\sum_{k}q_{lk}$ for each I and ${\;Q}^{T}Q=K$. M represents the nearest neighbours.

### 3.2 Diffusion maps (DM)

It is one of the global non-linear techniques of dimensionality reduction. Diffusion Maps were prepared by constructing a Markov random stroll on the data graph. A calculation of the data point's proximity is obtained by performing the random stroll for such a number of time steps. This so, diffusion distance is calculated using this method. The pair-wise diffusion distances are preserved as much as feasible in a low-dimensional set of data [30]. The first step is to construct a data graph. The Gaussian kernel function is used to calculate the weight of graph edges, resulting in a matrix.

(3) $$a_{kl}=e^{-\frac{{\left\|y_{k}-y_{l}\right\|}^{2}}{{2\sigma }^{2}}}$$

Where σ indicates the Gaussian variance, after that, the matrix a is normalized such that all rows stack up to one. So, the matrix $b^{\left(1\right)}$ is,

(4) $$b_{kl}^{\left(1\right)}=\frac{a_{kl}}{\sum_{g}a_{kg}}$$

Although diffusion maps derived through dynamical system theory, that resulting matrix $b^{\left(1\right)}$ is a markov matrix. The markov matrix describes a dynamical process forward transition probability matrix. $b^{\left(1\right)}$ is transition of one point to another point of data. So, probability matrix is $b^{\left(t\right)}$ is given by ${\left(b^{\left(1\right)}\right)}^{t}$. It is also defined as the diffusion distance.

(5) $$E^{t}\left(y_{k},y_{l}\right)=\sum_{g}\frac{{\left(b_{kg}^{\left(t\right)}-b_{\mathrm{lg}}^{\left(t\right)}\right)}^{2}}{\Psi {\left(y_{g}\right)}^{\left(0\right)}}$$

(6) $$\Psi {\left(y_{g}\right)}^{\left(0\right)}=\frac{v_{k}}{\sum_{l}v_{l}}$$

Where $v_{k}$ represents the Node degree, $y_{k}$ defined by $v_{k}=\sum_{l}b_{kl}$. The diffusion distance equation ${\;E}^{t}\left(y_{k},y_{l}\right)$, pairs of data points with such a large forward transfer. Probabilities have quite a small diffusion gap. As a result, the diffusion distance is more noise. That noise called geodesic distance. The ‘h’ nontrivial principal Eigen vector of Eigen problem forms the low dimensional representation. ‘S’ that preserves the diffusion distance

(7) $$E^{t}\left(S\right)=\lambda S$$

Where λ = 1 and its Eigen vector m_1,_ low dimensional representation ‘S’ is given by,

(8) $$S=\left\{\lambda_{2}m_{2\;},\lambda_{3}m_{3},\ldots \ldots \ldots \ldots \ldots \ldots \lambda_{h+1}m_{h+1}\right\}$$

**Fig. 3:**
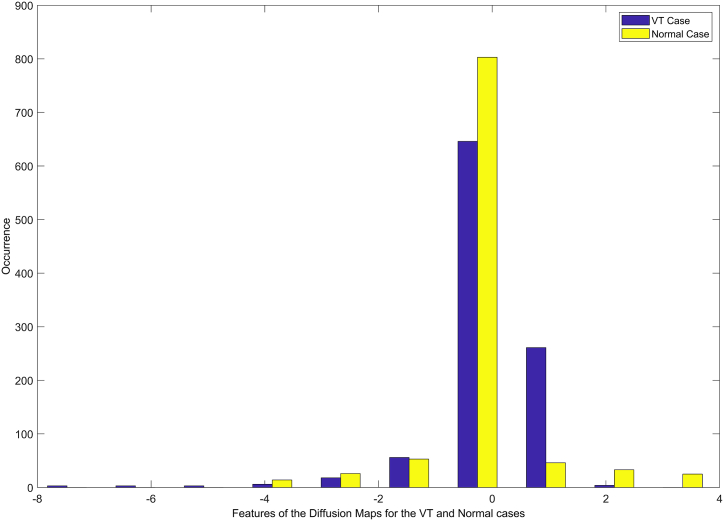
Histogram difference plot with diffusion maps features for VT and normal cases.

Where h represents the principal Eigenvectors, Fig. 3 illustrates the histogram of the different evaluations of diffusion map features for VT, PVC, ST, and normal cases. It is observed from Fig. 3 that the histogram for VT cases is non-Gaussian and skewed when compared to normal cases. The overlapping nature of the histogram variables is also clearly indicated in Fig. 3.

### 3.3 Laplacian Eigenmaps (LE)

It will be more relevant to the locally linear embedding technique in that the conservation of the local properties of the manifold is prioritized, allowing Eigenmaps to locate the lower-dimensional datasets conveniently. The pairwise distances between neighbours are used to evaluate local properties. They typically simulate a low-dimensional approximation of a specific dataset where the distances between datasets are diminished [32]. The LE algorithm begins by building its neighbourhood data matrix 'S' where each data points $g_{n}$ are linked toward its 'm' nearest neighbours. The weight of an edge is evaluated through using Gaussian kernel function $a_{nt}=e^{\frac{{-\left\|g_{n}-g_{t}\right\|}^{2}}{{2\sigma }^{2}}}$ for all points $g_{n}$ and $g_{t}$ in graph 'S' that are connected by an edge, where, σ is the Gaussian variance and resulting in A, a sparse adjacency matrix. The cost function that is reduced in the computation of its low dimensional projections $f_{n}$ is [30],

(9) $$ø\left(F\right)=\sum_{nt}{\left(f_{n}-f_{t}\right)}^{2}a_{nt}$$

Shorter gaps in between data points $g_{n}$ and $g_{t}$ with huge weights $a_{nt}$ in the cost function. As a result, the cost function is heavily influenced by the distance between their low dimensional representations $f_{n}$ and ${\;f}_{t}$. It is possible to formulate the minimization problem as an Eigen problem by computing the degree matrix 'P' and Laplacian graph 'E' of graph A. That row amount of A, That is

(10) $$h_{nn}=\sum_{t}a_{nt}$$

E=P−A is used to measure the Laplacian graph E. Therefore, equation (9) rewritten as,

(11) $$\emptyset \left(F\right)=2F^{\prime }EF$$

As a result, minimization of ∅(F) becomes proportional to minimization of $F^{\prime }EF$. So, it is solved the eigen vector problems. The low-dimensional data representation 'F' is formed by the 'l' eigen vectors $x_{i}$, which correspond to the smallest non zero eigenvalues.

(12) Ex=λPx

**Fig. 4:**
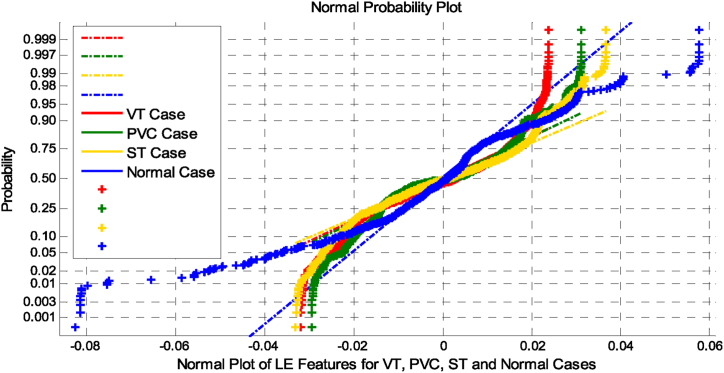
Normal plot evaluation of LE features for VT, ST, PVC and NSR

Fig. 4 represents the normal plot evaluation of LE features for VT, PVC, ST and Normal cases. Fig. 4 exhibits the overlapping nature of the class features among various classes. Therefore, to attain good classification accuracy, the selection of a classifier is more important. The statistical parameters such as Mean (μ), Standard Deviation (σ), Variance (σ^2^), Skewness (skew), Kurtosis (C), Pearson Correlation Coefficient (PCC), Approximate Entropy (ApEn), Renyi Entropy (ReEn) and Permutation Entropy (PeEn) along with different dimensionality reduction techniques for VT, PVC, ST and Normal Cases are presented in Table 2.

**Table 2:** Average statistical parameters at different dimensionality reduction techniques without CS and HSO feature selection for VT, PVC, ST and normal cases.

DR Techniques	Parameters	Arrhythmias			NSR
		VT	PVC	ST Change	
LLE	μ	0.1587	0.2119	0.1428	0.2318
	σ	0.8584	0.8338	0.8133	0.8641
	σ^2^	0.7603	0.7239	0.7118	0.7694
	skew	0.4965	0.5924	0.9676	1.0102
	C	6.9996	5.7416	5.3324	8.6129
	PCC	0.0043	−0.0002	0.0022	−0.0051
	ApEn	2.2860	2.0843	1.3388	0.4604
	ReEn	5.5215	5.5215	5.2983	3.1344
	PeEn	3.0093	3.0563	2.9028	1.9156
DM	μ	0.0110	0.0157	0.0285	0.0030
	σ	0.9206	0.9600	0.8618	0.9787
	σ^2^	0.8523	0.9220	0.7464	0.9580
	skew	−1.3373	−0.8230	−1.3108	−0.8284
	C	36.2101	6.18463	17.3949	9.4915
	PCC	0.0006	−0.0221	0.0075	0.0034
	ApEn	5.5215	5.5215	5.2983	5.2307
	ReEn	5.3487	5.4370	5.0034	5.2502
	PeEn	3.1248	3.0573	3.1556	3.1234
LE	μ	9.63E-06	2.73E-05	−8.24E-05	3.001E-05
	σ	0.0159	0.0160	0.0179	0.0189
	σ^2^	0.0003	0.0003	0.0003	0.0004
	skew	−0.0985	−0.0125	0.0860	0.0225
	C	−1.1649	−1.2692	−1.0400	0.7555
	PCC	0.0068	0.0148	0.0005	0.0034
	ApEn	5.5215	5.5215	5.2983	5.1940
	ReEn	2.7675	2.7507	2.7447	2.6299
	PeEn	3.1392	3.0861	3.1420	3.0548

The Normal plot curve is simulated as a non-linear with overlapping, as illustrated in Fig. 4. The CDF plot curve is simulated as unshaped, illustrated in Fig. 2. Table 2 summarizes the average statistical parameters for VT, PVC, ST and Normal Cases at various dimensionality reductions. In this, all statistical parameters and entropies between VT, PVC, ST, and Normal cases overlap, and there is the existence of non-linearity, as indicated by greater values of kurtosis and variance. These estimated parameters imply that further processing of the features will require a feature selection approach.

## 4 Feature selection

The dimensionally reduced ECG epoch values are fed into the feature selection using the Cuckoo Search algorithm (CS) and Harmonic Search Optimization (HSO) for selecting the features.

### 4.1 Cuckoo Search (CS)

Several Features in the ECG datasets can deceive the classifiers' prediction abilities since certain features can lead to incorrect classification. In order to enhance the classification accuracy, feature selection methods may be employed to choose informative features. The Cuckoo-Search is a well-known metaheuristic and nature-inspired optimization algorithm. CS algorithm is simple, efficient, and suitable for determining the search of arbitrary paths. It can be used to solve any engineering design problems. Cuckoos set their eggs in the nests of other host birds of different species. Unless the host bird discovers that the eggs are not its own, it will either destroy the egg or abandon the nest entirely. Cuckoo eggs that look like host bird eggs have advanced as a result of this. The three specific terms of the CS algorithm can be outlined as follows [33].

a. Each Cuckoo bird lays one egg at a time and deposits those in a collection that is selected at random.
b. The best nests and the best eggs can be passed on to future generations.
c. The set of possible host nests is defined, and the host bird discovers the cuckoo's egg with the probability of ${\;f}_{a}\epsilon \left[0,1\right]$.

The basis CS algorithm is based on the above rules. Levy flight corresponds to the arbitrary flight characteristics of birds, and it is used to determine its next position $f_{i}^{\left(t+1\right)}$, using the existing position $f_{i}^{t}$ as a starting point.

(13) $$f_{i}^{\left(t+1\right)}=f_{i}^{t}+\gamma ⊕Leavy\;\beta$$

(14) $$Leavy\;∼\;u=t^{-\beta }$$

Where γ>0 denotes the step size of scaling factor. γ denotes step size and ⊕ denotes entry-wise multiplication. Here γ=1 and β=0.2 are arrived by trial and error method based on MSE values, it is indicating infinite variance with infinite mean.

### 4.2 Harmonic search optimization (HSO)

An unbounded optimal solution is written as follows when dealing with harmonic search optimization (HSO) via clustering [34].

(15) $$\min\;e\left(Y\right);\;Py_{j}\leq y_{j}\leq Wy_{j}$$

Where e(Y) represents the function of object, Y represents the decision variable, $y_{j}$ is the $j^{th}$ variable of decision. ${Py}_{j}$ and ${Wy}_{j}$ represents the lower and upper bounds of the $j^{th}$ variable of decision. To solve the typical harmonic search problem, following 5 steps mentioned below.

Step 1 The problem algorithms parameters are set their default values in this step the problem parameters $n,{Py}_{j},{Wy}_{j}$ are initialized. The Stopping Criterion (SC), Pitch Adjusting Rate (PAR), Harmonic Size (HS) and Harmonic Memory Considering Rate (HCR), or the highest number of improvisations $\left(V_{\max}\right)$, are the four additional algorithm parameters that are initialized.
Step 2 The memory for harmonic is set up. Genetic algorithms and Harmonic Search are similar. It's a population-based optimization algorithm in GA, but in HS, the population is called Harmonic Memory (HM), and it's built as a solution vector. The following is Harmonic Memory (HM) representation [35].

(16) $$Harmonic\;Memory\;\left(HM\right)=\left[\begin{array}{ccc} y_{1}^{1} & y_{2}^{1}\;\ldots & y_{n}^{1}\\ y_{1}^{2} & y_{2}^{2}\;\ldots & y_{n}^{2}\\ y_{1}^{HS} & y_{2}^{HS}\ldots & y_{n}^{HS} \end{array}\right]$$

(17) $$y_{i}^{j}=Py_{j}+\left({Wy}_{j}-{Py}_{j}\right)*r\left(\right)$$

Where $y_{i}^{j}$ denotes the $i^{th}$ and $j^{th}$ of the decision and solution vector. r denotes the r∈[0,1].

Step 3 There is now a new harmonic; the three principles of pitch adjustment, harmonic memory consideration, and randomization are used to improve a novel harmonic vector.

(18) $$y^{new}=\left(y_{1}^{new},y_{2}^{new},\ldots ..y_{n}^{new}\right)$$

The PAR determines the probability of pitch change, while the harmonic memory consideration rate determines the probability of harmonic consideration.

(19) $$y_{j}^{new}=y_{j}^{new}\sum y_{j}^{1},y_{j}^{2},\ldots \ldots y_{j}^{HS}$$

(20) $$y_{j}^{new}={Py}_{j}+\left({Wy}_{j}-{Py}_{j}\right)*r\left(\right)$$

(21) $$y_{j}^{new}=y_{j}^{new}\pm bw*r\left(\right)$$

Where, $y_{j}^{new}$ represents the $j^{th}$ variable $y_{j}^{new}$ of normal harmonic vector, bw denotes the bandwidth.

Step 4 The HM has been refreshed. If the new and updated harmonic vector outperforms the worst harmonic vector in terms of objective function value, the improved harmonic vector effectively triumphs.
Step 5 The condition for stopping has been meticulously checked. If the stopping condition is met, the iteration is finished; if not, step 3 and 4 are continued.

Table 3 displays the average statistical parameters using different dimensionality reduction techniques with CS and HSO feature selection for VT, PVC, ST, and Normal cases. Table 3 shows that after CS and HSO feature selection, overlapping features are eliminated. Despite the existence of non-linearity, the improved feature is still used to provide superior segmentation by a select group of classifiers.

**Table 3:** Average statistical parameters at different dimensionality reduction techniques with CS and HSO feature selection for VT, PVC, ST and normal cases.

DR Techniques	With CS feature selection					With HSO feature selection			
	Parameters	Arrhythmias			NSR	Arrhythmias			NSR
		VT	PVC	ST Change		VT	PVC	ST Change	
LLE	μ	0.8993	0.8987	0.9039	0.8962	0.9297	0.9038	0.9018	0.8821
	σ	0.0298	0.0277	0.0320	0.0361	0.0603	0.0294	0.0367	0.0016
	σ^2^	0.0009	0.0008	0.0010	0.0013	0.0036	0.0010	0.0013	2.57E-06
	skew	0.2491	0.1544	0.0777	0.7223	1.2891	−0.1010	0.3167	−3.0147
	C	−1.3581	−1.1402	−1.5476	−0.8045	2.0426	−1.4587	−1.5312	12.0103
	PCC	0.0120	−0.0504	−0.0541	−0.0803	−0.0506	−0.0378	0.0426	0.0186
	ApEn	4.7123	4.4965	4.6779	5.0334	4.2282	4.5948	4.3031	7.8273
	ReEn	7.6208	7.6194	7.6312	7.6145	7.6905	7.6307	7.6268	7.5812
	PeEn	3.1137	3.1108	3.1315	2.7065	2.9838	3.1111	3.0004	3.1766
DM	μ	0.9104	0.9289	0.9178	0.8965	0.9299	0.9179	0.9222	0.8989
	σ	0.0250	0.0252	0.0190	0.0286	0.0321	0.0273	0.0154	0.0293
	σ^2^	0.0006	0.0006	0.0004	0.0008	0.0011	0.0008	0.0003	0.0010
	skew	−0.3435	−1.3893	−0.9342	0.4179	3.8391	−0.7003	−1.5644	0.2488
	C	−0.4929	0.8718	0.8063	−1.0273	−0.2556	−0.6732	3.2809	−1.1936
	PCC	0.0148	−0.1342	0.0211	−0.0016	−0.0230	0.0171	−0.0859	0.0176
	ApEn	7.8320	7.8320	7.7704	7.8163	7.8336	7.8320	7.8320	7.8312
	ReEn	7.6449	7.6851	7.6608	7.6145	7.6878	7.6614	7.6704	7.6200
	PeEn	3.1663	3.1583	3.1712	3.1723	3.1404	3.1677	3.1120	3.1746
LE	μ	0.8826	0.8826	0.8826	0.8827	0.8826	0.8826	0.8830	0.8826
	σ	6.21E-05	6.04E-05	9.17E-05	0.0002	7.59E-05	6.26E-05	0.0002	0.0001
	σ^2^	3.86E-09	3.69E-09	8.41E-09	2.61E-08	5.80E-09	3.95E-09	2.11E-08	1.23E-08
	skew	0.5229	0.7141	0.5221	−1.2103	0.1143	−0.0111	−0.5656	−0.0526
	C	1.2838	2.0314	0.6471	2.0908	0.8867	4.6052	0.7646	0.2582
	PCC	0.0111	−0.0019	0.0300	0.0338	0.0060	0.0026	−0.0041	0.0293
	ApEn	7.8092	7.8170	7.7667	7.7832	7.8155	7.8179	7.8124	7.8170
	ReEn	7.5822	7.5822	7.5823	7.5827	7.5845	7.5823	7.5832	7.5823
	PeEn	3.1719	3.1665	3.1688	3.1725	3.1312	3.1348	3.1310	3.1723

## 5 Classifiers for different cardiac arrhythmias detection

Dimensionally reduced ECG epoch values and epoch values from CS and HSO feature selection are fed into classifiers for detecting different cardiac arrhythmias. The classifiers used include Gaussian Mixture Model (GMM), Expectation Maximization (EM), Non-linear Regression (NLR), Logistic Regression (LR), Bayesian Linear Discriminant Analysis (BDLC), Detrended Fluctuation Analysis (Detrended FA) and Firefly.

### 5.1 Gaussian Mixture Model (GMM)

A Gaussian mixture model represents a probability density function (PDF) of its random variable, $\sum s^{g}$. Where ‘n’ is Gaussian distributions given by,

(22) $$V\left(q|\;r\right)=\sum_{k=1}^{n}\beta_{k\;}V\left(q|\;t\right)$$

Where q indicates the data vector, r represents the mixture model, $\beta_{k\;}$ indicates the component weight of k. k = 1, ….n and component density is,

(23) $$V\left(q|\;t\right)=\frac{{\left|\sum_{k}\right|}^{-1/2}}{{\left(2\pi \right)}^{d/2}}\exp\left\{-\frac{1}{2}\;{\left(q-\mu_{k}\right)}^{l}\;\frac{1}{\sum_{k}}\;\left(q-\mu_{k}\right)\right\}$$

Where $\mu_{k}$ represents the mean and co-variance of matrix, $\sum_{k}$ indicates the mixture weights, it is satisfy the condition that $\sum_{k=1}^{n}\beta_{k\;}=1$. GMM considerations are most often obtained from the training data and to use the Expectation maximization (EM) iterative algorithm, tough map estimation is used sometimes. The resulting GMM is validated using the mixture weights, covariance matrices and mean vectors from many of the parameter densities. The following terminology is used to represent the parameter collectively [36].

(24) $$r=\left\{\beta_{k}\;\mu_{k},\sum_{k}\right\}\;k=1,\ldots ..n$$

Covariance matrices $\sum_{k}$ can also be a complete class otherwise restricted such that they are diagonal. The model configuration in GMM is determined by the total quantity of data available to estimate their GMM parameters. It is important to note that even though the features aren't statistically independent, complete covariance matrices aren't needed so because Gaussian components aren't explicitly working together to simulate the feature density. The successful presence of linear combination with diagonal covariance premise Gaussian is used to model the association between the function vector components. The sequence of n complete covariance matrix Gaussian can be easily obtained by using a greater set of diagonal covariance Gaussian. Maximum probability parameter estimate is used for the estimation. If the vector is considered to be different, its GMM probability is described as follows for a given sequence of G training vectors.

(25) $$Q=\left\{q_{1},\ldots \ldots \ldots q_{G}\right\}$$

(26) $$V\left(Q|r\right)=\prod_{g=1}^{G}V\left(q_{g}|r\right)$$

The above equations show that ‘r’ is a non-linear parameter and maximization (direct) is not possible. However, the optimal case of the expectation maximization (EM) algorithm can be used. The maximization likelihood parameter can be easily obtained iteratively. The EM algorithm most basic approach is to take via an original model ‘r’ but instead estimate the new model $\bar{r}$, so that

(27) $$V\left(Q|\bar{r}\right)\geq V\left(Q\geq r\right)$$

The weight of mixture is given by

(28) $$\beta_{k}=\frac{1}{G}\;\sum_{g=1}^{G}V_{s}\left(k|q_{g},r\right)$$

(29) $${\bar{\mu }}_{k}=\frac{\sum_{g=1}^{G}V_{s}\left(k|q_{g},r\right)q_{g}}{\sum_{g=1}^{G}V_{s}\left(k|q_{g},r\right)}$$

The diagonal covariance's are,

(30) $${\bar{\sigma }}_{i}^{2}=\frac{\sum_{g=1}^{G}V_{s}\left(k|q_{g},r\right){q_{g}}^{2}}{\sum_{g=1}^{G}V_{s}\left(k|q_{g},r\right)}-\;{\bar{\mu }}_{k}^{2}$$

$\sigma_{i}^{2}$ , $q_{g}$ and $\mu_{k}$ – it is refers to arbitrary vector elements. Finally $V_{s}\left(k|q_{g},r\right)$ is given by following equation,

(31) $$V_{s}\left(k|q_{g},r\right)=\frac{\beta_{k}V\left(q_{g}|\mu_{k}\sum_{k}\right)}{\sum_{O=1}^{n}\beta_{0}V\left(q_{g}|\mu_{0}\sum_{0}\right)}$$

### 5.2 Expectation maximization (EM)

Expectation maximization (EM) is a mathematical method for optimizing dynamic likelihoods and solving problems with missing results. In general, (i) Expectation step (E) and (ii) Maximization step (M) are two steps of the EM algorithm [37].

(i) Expectation Step (E):

Define data ${\;g}_{1}$, which contains a parameters approximation and observed data; that estimated value can be quickly calculated initially. The estimated value of $g_{1}$ is calculated as follows for a given measurement $s_{1}$ and looking at the current approximation of its variable.

(32) $$g_{1}^{\left[m+1\right]}=E\left[g_{1}|s_{1},b^{m}\right]$$

(ii) Maximization Step (E):

We will use data that has been actually determined to calculate the parameter's maximum likelihood approximation after the expectation stage. An collection of unit vectors is described as $\bar{Q}$. Considering ${{\;g}_{i}}^{\prime \prime }G$, therefore likelihood of G is given by

(33) $$b\left(G|\mu ,M,\mu ,m\right)=b(g_{l}\ldots \ldots ..g_{t}\left|\mu ,k\;g_{l}\;\ldots \ldots ,g\right|\mu ,K\;g_{l},\ldots ..g|\mu ,K$$

(34) $$=\prod_{l=1}^{t}f\left(g_{l}|\mu ,k\right)\prod_{l=1}^{t}q_{r}\left(k\right)e^{{k\mu }^{H}g_{l}}$$

The above equation is likelihood can also be given as

(35) $$E\left(K|\mu ,k\right)=\ln\;b\;\left(K|\mu ,k\right)=t\;\ln\;q_{r}\left(k\right)+k\mu^{H}y$$

(36) $$y=\sum_{l}g_{l}$$

We'll need to use the Lagrange operator ‘v’ to optimize the equation to get the likelihood parameters &k. The modified equation can then be written as follows,

(37) $$E\left(\mu ,v,k,K\right)=t\;\ln\;q_{r}\left(k\right)+k\mu^{H}y+v\left(1-\mu^{H}\mu \right)$$

The parameter constraints are obtained by deriving the above equation with respect to μ,v&k and equating these to 0. Therefore,

(38) $$\mu =\frac{\hat{k}}{2\hat{v}}y$$

(39) $$\mu^{H}\hat{\mu \;}=1$$

(40) $$\frac{{tq}^{\prime }\left(\hat{k}\right)}{q_{r}\left(\hat{k}\right)}=-{\hat{\mu }}^{H}y$$

Since both the observed data and the present approximation of its model parameters were given, threshold data will be first calculated within the expectation stage. To achieve this, the conditional expectation has been used, which illustrates the terminology preference the likelihood function was maximized in the Maximization-step values obtained underneath the premise that even the threshold statistics were known. In place of its real threshold statistics, the expectation steps for the calculation of the missing data are used.

### 5.3 Non linear regression (NLR)

Non-linear processes are sometimes required to identify real-world phenomena where linear models are insufficient. All such regression models would have some basic framework, that is,

(41) s=g(a)

Non-linear regression (NLR) may provide a smoother line unless the variable $\prime s^{\prime }$ is random, while linear regression (LR) may equate any two parameters with such a straight line in the pattern of s=ja+d. So, the main target of NLR would be to reduce the number of its squares, which represents the degree to which an individual findings vary from the mean of dataset's [38]. Therefore, the NLR function $\prime s^{\prime }$ is defined as follows [39],

(42) s∼g(a,γ)

It connects a set of independent variable (a) to the observed dependent variable (s) and in component of its vector parameters, the function 'g' is non-linear, but even then it is arbitrary. For example, to achieve error free classification outcomes, the following NLR model is represented by non-linear function of $\prime \gamma^{\prime }$.

(43) $$g\left(a,\gamma \right)=\frac{\gamma_{1}a}{\gamma_{2}+a}$$

The model of non-linear regression (NLR) is written as follows,

(44) $$s_{m}=g\left(a_{m},\gamma \right)+p_{m1}$$

Where 'g' denotes the function of expectation and $a_{m}$ denotes the independent variable. Each of the variants of the expectation function 'g' for non-linear models should be dependent at least one of the variables. In a non-linear model, γ is used as the parameters. J is the number of parameters that are being considered. Where analysing a set of data, consider vector $a_{m}$, where m=1,2,3,…M and it is fixed to concentrate the expected response's dependence on. The $m^{th}$ element of the M-vector λ(γ) is now formed.

(45) $$\lambda_{m}\left(\gamma \right)=g\left(a_{m},\gamma \right);\;m=1,\ldots \ldots M$$

The non-linear regression model is mathematically written as follows:

(46) S=λ(γ)+p

Where p denotes the spherical normal distribution, it is written as follows,

(47) $$D\left(p\right)=0;Var\;\left(p\right)=D\left(pp^{\prime }\right)=\sigma^{2}J$$

In geometrical manner, the least squares values can be easily sought. m=1,……M for a given data vector 's', an expectation function $g\left(a_{m},\gamma \right)$ and set of design vector $a_{m}$.

### 5.4 Logistic Regression (LR)

Logistic Regression (LR) is among the most widely used classifiers. To approximate the value of a statistical variable 'g' in the future when g∈[0,1], is ‘0’ means negative class and ‘1’ means positive class for a binary classification problem [35]. The single outcome vector, $g_{m}\left(m=1,2,3,\ldots ..n\right)$, is coded ‘1’ for a specific probability $s_{m}$ and ‘0’ for a specific probability $1-s_{m}$. The $s_{m}$ differs in such a statistical context, along with ${\;f}_{m}$, as a function of certain parameters and has been represented as,

(48) $$Z\left[g_{m}|f_{m},\gamma \right]=s_{m}=\frac{e^{f_{m}\gamma }}{1+e^{f_{m}\gamma }}$$

Where γ is a parameters of vectors, with the implication that $f_{m0}=1\text{.}$ As a result, the logistic transformation is defined as the logarithm of the positive outcome odds, and it is represented as follows [40],

(49) $$k_{m}=\ln\left[\frac{s_{m}}{1-s_{m}}\right]=f_{m}\gamma$$

The logistic function is written as follows in matrix form: the standardized log-likelihood and the loss function negative likelihood are calculated using the following formulas,

(50) k=Fγ

(51) $$\ln\;H\left(\gamma \right)=\sum_{m=1}^{N}\left(g_{m}\;\ln\;s_{m}+\left(1-g_{m}\right)\ln\left(1-s_{m}\right)\right)-\frac{\alpha }{2}{\left\|\gamma \right\|}^{2}$$

(52) $$DEV\left(\hat{\gamma }\right)=-2\;\ln\;H\left(\gamma \right)$$

The loss function also known as the deviance (DEV). The regularization expression $\frac{\alpha }{2}{\left\|\gamma \right\|}^{2}$ is applied in order to achieve a greater generalization.

### 5.5 Bayesian discriminant linear Classifier (BDLC)

The Bayes determination rule is fully reliant on the BDLC classifier to decrease the probability error. In a function vector 'g'; the class with the highest posterior probability is picked otherwise, if there are two classes 'm' and 'n', to pick class $\prime m^{\prime }$ if [41],

(53) $$s_{m}\left(g\right)-s_{n}\left(g\right)\geq M$$

With 'M' as the deciding threshold, the discriminant function $s_{m}\left(g\right)$ is defined as,

(54) $$s_{m}\left(g\right)=\ln\;J\left(m|g\right)$$

Any class observation is derived solely from the multivariate normal distribution. As a result of the Bayes principle, the covariance matrix for all categories is equivalent, and the discriminant function is given as follows,

(55) $$s_{m}\left(g\right)=-0.5{\left(g-\mu_{m}\right)}^{,}\frac{1}{\epsilon }\left(g-\mu_{m}\right)+\ln\;J\left(m\right)$$

Where, $\mu_{m}$ denotes the mean function vector for a given class 'm', ∈ denotes the matrix of covariance and J(m) denotes its prior probability for class 'm' [42]. The deciding boundary M is defined as follows, if the prior probabilities of all categories are assumed to be static. The separability of classes increases as the factor of $\frac{1}{\in }\left(\mu_{m}-\mu_{n}\right)$ increases.

(56) $${\left(g-\mu_{n}\right)}^{\prime }\frac{1}{\in }\left(g-\mu_{n}\right)-{\left(g-\mu_{m}\right)}^{\prime }\frac{1}{\epsilon }\;\left(f-\mu_{m}\right)$$

### 5.6 Detrended Fluctuation Analysis (detrended FA)

The Detrended FA, which is equivalent to the Hurst exponent study, is the result of a development in conventional fluctuation analysis correlation properties that can be calculated on a significant time scale basis in this case [43]. The random walk principle very significant in Detrended FA, a time series $\left(g_{m}\right)$, m=1,2,3,….Q with mean of 'g' description is defined as follows [44],

(57) $$G\left(p\right)=\sum_{m=1}^{p}\left(g_{m}-\left(g\right)\right)$$

The profile then divided into $Q_{u}=\left(Q/u\right)$ non overlapping segments, each with an equivalent of u is length of scale. The mean squared fluctuation function is expressed as follows for the Detrended FA approach.

(58) $$k^{2}\left(u\right)=\frac{1}{Q_{u}}\sum_{j=1}^{Q_{u}}{\left[G\left(\left(j-1\right)u\right)-G\left(ju\right)\right]}^{2}$$

Least square fitting is used to approximate a piecewise polynomial regression $x_{u}^{\left(y\right)}\left(p\right)$ within each section j. Now we'll look at the profile element, which has been detrended on a particular scale u. The fluctuation function on a particular scale u is now given by the variance of ${\tilde{G}}_{u}\left(p\right)$ expressed as follows,

(59) $${\tilde{G}}_{u}\left(p\right)=G\left(p\right)-\;x_{u}^{\left(y\right)}\left(p\right)$$

(60) $$k\left(u\right)={\left\{\frac{1}{Q}\sum_{p=1}^{Q}{\tilde{G}}_{u}^{2}\left(p\right)\right\}}^{\frac{1}{2}}$$

The trend-eliminated root mean square displacement is represented by equation (59), which must be determined with various scales of u.

### 5.7 Firefly

The firefly algorithm, which was invented by Yang [44], is used to simulate the flashing phenomenon of fireflies. The following hypotheses are taken in order to simulate the definition. The fireflies are the entire same genus. Fireflies that really are brighter draw more attention than fireflies that are less vivid. The attraction between the fireflies reduces as the distance between them rises. Since no firefly is sharper than another, the firefly would travel at random. The landscapes of a given objective feature heavily influence a firefly's light. The brightness ‘k’ is represented as,

(61) k(x)αE(x)

(62) $$k\left(p\right)=\left(k_{0}\right)e^{-\beta p^{2}}$$

Where $k_{0}$ indicates the light of actual density and β represents the coefficient of absorption

Attractiveness α as follows,

(63) $$\alpha \left(p\right)=\alpha_{0}\left(e^{-\beta p^{2}}\right)$$

Where p represents the gap between two fireflies, $\alpha_{0}$ indicates the attractiveness at condition p = 0. Separation between $p_{mn}$ of two fireflies is $z_{m}$ and $z_{n}$ is estimated as

(64) $$p_{mn}=\left\|z_{m}-z_{n}\right\|=\sqrt{\sum_{j=1}^{d}\left(z_{m,j}-z_{n,j}\right)}$$

Where $z_{m,j}$ represents the jth component of coordinate spatial of ${\;z}_{m}$. Its exact behaviour of the firefly (m) attracted to an even much brighter firefly (n), it is determined as follows

(65) $$z_{m}=z_{m}+\gamma \left(rand-\frac{1}{2}\right)+\alpha_{0}e^{-\beta p_{mn}^{2}}\left(z_{n}-z_{m}\right)$$

Where r indicates the random parameter [0, 1] and rand represents the Gaussian distribution [0, 1].

(66) $$z_{m}=z_{m}+\alpha_{\min}+\left(\alpha_{0}-\alpha_{\min}\right)e^{-\beta p_{mn}^{2}}+\gamma *\left(rand-\frac{1}{2}\right)*scale$$

## 6 Training and testing

Dimensionally reduced ECG epoch values and epoch values from CS and HSO feature selection are used separately for both training and testing. This same training was developed using such a regressive approach, and the classifier MSE values were condensed to the least one. All of the classifiers were trained with an MSE of zero training error. The kinds of cross-validation approach used in this work were K fold. The dataset is primarily segmented into K equal-sized points. For the training of the classifiers, K−1 sets are used for performance evaluation in each step, and then the remaining step is used. The validation cycle is continued for a total of K times. The performance of the classifier calculation is evaluated using the K results. The value of K in our work is set to 10. As a result, 90 % of the epochs were utilized for training, while just 10 % was used for testing. In this work, epochs of one class are equally distributed across the folds. Based on the low MSE values attained in the CS feature selection methods for different classifiers, which will be a marker for good classifier performance.

### 6.1 Mean Square Error (MSE)

The sum of its squared errors, or the average squared variance between both the predicted and real value, is calculated by Mean Square Error (MSE). Because of randomness, MSE is almost always purely positive rather than zero. The monitoring of the MSE is used to observe the training and testing process [45],

(67) $$\mathrm{MSE}=\frac{1}{M}\sum_{k=1}^{M}{\left(G_{k}-S_{l}\right)}^{2}$$

Where $G_{k}$ indicates the value of observed at particular time, $S_{l}$ represents the value of target at l. In VT case without CS and HSO feature selection, $S_{l}$ ranges from 1to24 with M equal to 2167 epochs. In PVC case without CS and HSO feature selection, $S_{l}$ ranges from 1to32 with M equal to 2889 epochs. In ST case without CS and HSO feature selection, $S_{l}$ ranges from 1to56, with M equal to 4200 epochs. In NSR case without CS and HSO feature selection, $S_{l}$ ranges from 1to36, with M equal to 7088 epochs. In VT case with CS and HSO feature selection; ${\;S}_{l}$ ranges from 1to24, with M equal to 333 epochs. In PVC case with CS and HSO feature selection; $S_{l}$ ranges from 1to32, with M equal to 444 epochs. In ST case with CS and HSO feature selection, $S_{l}$ ranges from 1to56, with M equal to 778 epochs. In NSR case with CS and HSO feature selection; ${\;S}_{l}$ ranges from 1to36, with M equal to 1406 epochs.

Table 4 displays the average MSE values and confusion matrix for the VT vs. NSR, PVC vs. NSR, and ST vs. NSR classes using different classifiers without CS and HSO feature selection for various dimensionality reduction techniques. Table 5 exhibits the average MSE values and confusion matrix for VT vs. NSR, PVC vs. NSR, and ST vs. NSR classes for different classifiers with CS feature selection in different dimensionality reduction techniques. Table 6 exhibits the average MSE values and confusion matrix for VT vs. NSR, PVC vs. NSR, and ST vs. NSR classes for different classifiers with HSO feature selection in different dimensionality reduction techniques. Average MSE values and confusion matrix for VT vs. NSR, PVC vs. NSR, and ST vs. NSR classes with GSO and Adam hyperparameter tuning based on different classifiers with CS feature selection for different dimensionality reduction techniques are shown in Table 7. Table 8 exhibits the Average MSE values and confusion matrix for VT vs. NSR, PVC vs. NSR, and ST vs. NSR classes with GSO and Adam hyperparameter tuning based on different classifiers with HSO feature selection for different dimensionality reduction techniques.

**Table 4:** Average MSE values and confusion matrix for VT vs. NSR, PVC vs. NSR, and ST vs. NSR classes with different classifiers without CS and HSO feature selection for different dimensionality reduction techniques.

DR Techniques	Classifiers	VT vs. NSR					PVC vs. NSR					ST vs. NSR				
		TP	TN	FP	FN	Average MSE	TP	TN	FP	FN	Average MSE	TP	TN	FP	FN	Average MSE
LLE	GMM	1275	4080	3008	892	0.000074	1700	4080	3008	1189	0.000074	2986	4080	3008	1214	0.000053
	EM	1253	3762	3326	914	0.000209	1715	3762	3326	1174	0.000206	2942	3762	3326	1258	0.000187
	NLR	1185	3839	3249	982	0.000211	1625	3839	3249	1264	0.000163	2286	3839	3249	1914	0.000218
	LR	1150	3959	3129	1017	0.000208	1806	3959	3129	1083	0.000083	2330	3959	3129	1870	0.000116
	BDLC	1127	3781	3307	1040	0.000345	1602	3781	3307	1287	0.000205	2713	3781	3307	1487	0.000169
	Detrended FA	1111	3839	3249	1056	0.000366	1629	3839	3249	1260	0.000162	2229	3839	3249	1971	0.000266
	Firefly	1242	4010	3078	925	0.000088	1591	4010	3078	1298	0.000119	2838	4010	3078	1362	0.000068
DM	GMM	1185	3904	3184	982	0.000170	1553	3904	3184	1336	0.000200	2494	3904	3184	1706	0.000106
	EM	1165	3885	3203	1002	0.000213	1553	3885	3203	1336	0.000106	3011	3885	3203	1189	0.000098
	NLR	1121	3894	3194	1046	0.000299	1584	3894	3194	1305	0.000163	2592	3894	3194	1608	0.000108
	LR	1111	3881	3207	1056	0.000344	1473	3881	3207	1416	0.000356	2258	3881	3207	1942	0.000219
	BDLC	1133	3857	3231	1034	0.000295	1533	3857	3231	1356	0.000258	2625	3857	3231	1575	0.000133
	Detrended FA	1105	3615	3473	1062	0.000529	1700	3615	3473	1189	0.000300	2229	3615	3473	1971	0.000418
	Firefly	1174	4017	3071	993	0.000159	1648	4017	3071	1241	0.000089	2863	4017	3071	1337	0.000065
LE	GMM	1139	3810	3278	1028	0.000309	1685	3810	3278	1204	0.000164	2680	3810	3278	1520	0.000154
	EM	1127	4054	3034	1040	0.000241	1602	4054	3034	1287	0.000066	2538	4054	3034	1662	0.000073
	NLR	1111	4024	3064	1056	0.000298	1553	4024	3064	1336	0.000102	2319	4024	3064	1881	0.000111
	LR	1133	3650	3438	1034	0.000390	1553	3650	3438	1336	0.000328	2740	3650	3438	1460	0.000224
	BDLC	1116	3878	3210	1051	0.000336	1625	3878	3210	1264	0.000144	2647	3878	3210	1553	0.000121
	Detrended FA	1105	3857	3231	1062	0.000370	1473	3857	3231	1416	0.000370	2308	3857	3231	1892	0.000183
	Firefly	1121	4054	3034	1046	0.000262	1580	4054	3034	1309	0.000139	2740	4054	3034	1460	0.000065

**Table 5:** Average MSE values and confusion matrix for VT vs. NSR, PVC vs. NSR, and ST vs. NSR classes for different classifiers with CS feature selection in different dimensionality reduction techniques.

DR Techniques	Classifiers	VT vs. NSR					PVC vs. NSR					ST vs. NSR				
		TP	TN	FP	FN	Average MSE	TP	TN	FP	FN	Average MSE	TP	TN	FP	FN	Average MSE
LLE	GMM	247	1044	362	86	0.000021	271	1044	362	173	0.000042	565	1044	362	213	0.000022
	EM	212	981	425	121	0.000043	296	981	425	148	0.000039	543	981	425	235	0.000034
	NLR	214	908	498	119	0.000050	302	908	498	142	0.000044	533	908	498	245	0.000043
	LR	219	917	489	114	0.000047	253	917	489	191	0.000068	470	917	489	308	0.000056
	BDLC	188	809	597	145	0.000088	276	809	597	168	0.000068	478	809	597	300	0.000069
	Detrended FA	200	813	593	133	0.000071	253	813	593	191	0.000081	467	813	593	311	0.000071
	Firefly	232	1102	304	101	0.000025	311	1102	304	133	0.000025	610	1102	304	168	0.000017
DM	GMM	235	956	450	98	0.000036	282	956	450	162	0.000046	515	956	450	263	0.000042
	EM	212	917	489	121	0.000050	282	917	489	162	0.000050	513	917	489	265	0.000047
	NLR	190	938	468	143	0.000064	293	938	468	151	0.000045	529	938	468	249	0.000042
	LR	190	807	599	143	0.000082	293	807	599	151	0.000063	464	807	599	314	0.000073
	BDLC	225	815	591	108	0.000057	290	815	591	154	0.000061	503	815	591	275	0.000061
	Detrended FA	200	828	578	133	0.000068	264	828	578	180	0.000070	464	828	578	314	0.000069
	Firefly	241	1022	384	92	0.000024	310	1022	384	134	0.000028	529	1022	384	249	0.000031
LE	GMM	232	850	556	101	0.000049	276	850	556	168	0.000061	474	850	556	304	0.000063
	EM	208	873	533	125	0.000057	278	873	533	167	0.000057	519	873	533	259	0.000051
	NLR	192	879	527	141	0.000067	274	879	527	170	0.000058	513	879	527	265	0.000051
	LR	188	839	567	145	0.000083	253	839	567	191	0.000077	462	839	567	316	0.000068
	BDLC	207	868	538	126	0.000059	253	868	538	191	0.000074	523	868	538	255	0.000051
	Detrended FA	190	873	533	143	0.000072	253	873	533	191	0.000073	478	873	533	300	0.000059
	Firefly	191	1005	401	142	0.000053	256	1005	401	188	0.000052	565	1005	401	213	0.000025

**Table 6:** Average MSE values and confusion matrix for VT vs. NSR, PVC vs. NSR, and ST vs. NSR classes for different classifiers with HSO feature selection in different dimensionality reduction techniques.

DR Techniques	Classifiers	VT vs. NSR					PVC vs. NSR					ST vs. NSR				
		TP	TN	FP	FN	Average MSE	TP	TN	FP	FN	Average MSE	TP	TN	FP	FN	Average MSE
LLE	GMM	309	1194	212	24	0.000004	428	1194	212	16	0.000004	762	1194	212	16	0.000004
	EM	276	1044	362	57	0.000015	412	1044	362	32	0.000011	750	1044	362	28	0.000011
	NLR	231	1091	315	102	0.000026	348	1091	315	96	0.000017	490	1091	315	288	0.000036
	LR	231	864	542	102	0.000048	293	864	542	151	0.000054	630	864	542	148	0.000037
	BDLC	229	981	425	104	0.000035	296	981	425	148	0.000039	529	981	425	249	0.000037
	Detrended FA	199	1005	401	134	0.000047	302	1005	401	142	0.000033	533	1005	401	245	0.000032
	Firefly	242	1102	304	91	0.000020	378	1102	304	66	0.000012	543	1102	304	235	0.000025
DM	GMM	302	1120	286	31	0.000008	430	1120	286	14	0.000008	754	1120	286	24	0.000008
	EM	206	1139	267	127	0.000036	416	1139	267	28	0.000007	746	1139	267	32	0.000007
	NLR	205	1142	264	128	0.000037	318	1142	264	126	0.000019	577	1142	264	201	0.000017
	LR	212	1022	384	121	0.000038	293	1022	384	151	0.000035	529	1022	384	249	0.000031
	BDLC	288	1069	337	45	0.000012	290	1069	337	154	0.000033	526	1069	337	252	0.000030
	Detrended FA	242	1084	322	91	0.000021	276	1084	322	168	0.000038	499	1084	322	279	0.000034
	Firefly	284	1135	271	49	0.000010	338	1135	271	106	0.000017	620	1135	271	158	0.000014
LE	GMM	264	1113	293	69	0.000016	416	1113	293	28	0.000008	746	1113	293	32	0.000008
	EM	269	1091	315	64	0.000016	428	1091	315	16	0.000009	758	1091	315	20	0.000009
	NLR	271	1084	322	62	0.000015	397	1084	322	47	0.000011	474	1084	322	304	0.000040
	LR	226	926	480	107	0.000043	317	926	480	127	0.000037	519	926	480	259	0.000045
	BDLC	232	1005	401	101	0.000030	276	1005	401	168	0.000042	513	1005	401	265	0.000037
	Detrended FA	228	931	475	105	0.000041	278	931	475	167	0.000051	556	931	475	222	0.000036
	Firefly	239	990	416	94	0.000029	330	990	416	114	0.000026	543	990	416	235	0.000033

**Table 7:** Average MSE values and confusion matrix for VT vs. NSR, PVC vs. NSR, and ST vs. NSR classes with GSO and Adam hyperparameter tuning based different classifiers with CS feature selection for different dimensionality reduction techniques.

CS features with GSO hyperparameter tuning																
DR Techniques	Classifiers	VT vs. NSR					PVC vs. NSR					ST vs. NSR				
		TP	TN	FP	FN	Average MSE	TP	TN	FP	FN	Average MSE	TP	TN	FP	FN	Average MSE
LLE	GMM	271	1194	212	62	0.000010	348	1194	212	96	0.000012	663	1194	212	115	0.000006
	EM	264	1150	256	69	0.000014	318	1150	256	126	0.000019	543	1150	256	235	0.000023
	BDLC	269	1139	267	64	0.000013	302	1139	267	142	0.000026	556	1139	267	222	0.000020
	Firefly	284	1219	187	49	0.000006	378	1219	187	66	0.000006	685	1219	187	93	0.000004
DM	GMM	227	1161	245	106	0.000024	318	1161	245	126	0.000018	577	1161	245	201	0.000016
	EM	245	1139	267	88	0.000018	342	1139	267	102	0.000016	529	1139	267	249	0.000026
	BDLC	220	1102	304	113	0.000032	389	1102	304	56	0.000010	526	1102	304	252	0.000029
	Firefly	276	1186	220	57	0.000009	345	1186	220	99	0.000013	624	1186	220	154	0.000011
LE	GMM	333	1406	0	0	0.000022	317	1091	315	127	0.000023	565	1091	315	213	0.000020
	EM	239	1091	315	94	0.000038	313	1084	322	131	0.000025	555	1084	322	223	0.000023
	BDLC	207	1084	322	126	0.000037	310	1139	267	134	0.000023	558	1139	267	220	0.000019
	Firefly	205	1139	267	128	0.000013	367	1161	245	77	0.000010	648	1161	245	130	0.000010

CS features with Adam hyperparameter tuning																
DR Techniques	Classifiers	VT vs. NSR					PVC vs. NSR					ST vs. NSR				
		TP	TN	FP	FN	Average MSE	TP	TN	FP	FN	Average MSE	TP	TN	FP	FN	Average MSE
LLE	GMM	291	1304	102	42	0.000002	391	1304	102	53	0.000002	711	1304	102	67	0.000001
	EM	270	1282	124	63	0.000007	385	1282	124	59	0.000003	697	1282	124	81	0.000002
	BDLC	250	1194	212	83	0.000014	365	1194	212	79	0.000009	610	1194	212	168	0.000012
	Firefly	264	1252	154	69	0.000009	375	1252	154	69	0.000005	632	1252	154	146	0.000008
DM	GMM	272	1230	176	61	0.000008	389	1230	176	56	0.000004	663	1230	176	115	0.000004
	EM	247	1216	190	86	0.000013	372	1216	190	72	0.000007	661	1216	190	117	0.000006
	BDLC	239	1172	234	94	0.000018	356	1172	234	88	0.000012	600	1172	234	178	0.000014
	Firefly	258	1201	205	75	0.000012	365	1201	205	79	0.000009	645	1201	205	133	0.000008
LE	GMM	271	1219	187	62	0.000009	375	1219	187	69	0.000006	665	1219	187	113	0.000006
	EM	275	1201	205	58	0.000008	370	1201	205	74	0.000008	656	1201	205	122	0.000007
	BDLC	264	1157	249	69	0.000013	360	1157	249	84	0.000012	630	1157	249	148	0.000012
	Firefly	267	1201	205	66	0.000010	363	1201	205	81	0.000009	640	1201	205	138	0.000009

**Table 8:** Average MSE values and confusion matrix for VT vs. NSR, PVC vs. NSR, and ST vs. NSR classes with GSO and Adam hyperparameter tuning based different classifiers with HSO feature selection for different dimensionality reduction techniques.

HSO features with GSO hyperparameter tuning																
DR Techniques	Classifiers	VT vs. NSR					PVC vs. NSR					ST vs. NSR				
		TP	TN	FP	FN	Average MSE	TP	TN	FP	FN	Average MSE	TP	TN	FP	FN	Average MSE
LLE	GMM	298	1267	139	35	0.000002	406	1267	139	38	0.000002	697	1267	139	81	0.000002
	EM	291	1230	176	42	0.000004	361	1230	176	83	0.000008	600	1230	176	178	0.000011
	BDLC	297	1216	190	36	0.000004	345	1216	190	99	0.000011	624	1216	190	154	0.000010
	Firefly	312	1318	88	21	0.000001	416	1318	88	28	0.000001	721	1318	88	57	0.000001
DM	GMM	265	1260	146	68	0.000009	363	1260	146	81	0.000007	661	1260	146	117	0.000005
	EM	264	1230	176	69	0.000010	375	1230	176	69	0.000006	630	1230	176	148	0.000008
	BDLC	253	1208	198	80	0.000013	393	1208	198	51	0.000004	620	1208	198	158	0.000010
	Firefly	300	1282	124	33	0.000002	405	1282	124	39	0.000001	689	1282	124	89	0.000002
LE	GMM	270	1201	205	63	0.000010	365	1201	205	79	0.000009	624	1201	205	154	0.000010
	EM	247	1172	234	86	0.000015	360	1172	234	84	0.000011	632	1172	234	146	0.000011
	BDLC	242	1225	181	91	0.000014	342	1225	181	102	0.000011	604	1225	181	174	0.000011
	Firefly	297	1238	168	36	0.000003	382	1238	168	62	0.000005	669	1238	168	109	0.000005

HSO features with Adam hyperparameter tuning																
DR Techniques	Classifiers	VT vs. NSR					PVC vs. NSR					ST vs. NSR				
		TP	TN	FP	FN	Average MSE	TP	TN	FP	FN	Average MSE	TP	TN	FP	FN	Average MSE
LLE	GMM	312	1399	7	21	0.00000032	423	1399	7	21	0.00000018	762	1399	7	16	0.00000002
	EM	298	1362	44	35	0.00000117	412	1362	44	32	0.00000045	711	1362	44	67	0.00000065
	BDLC	284	1384	22	49	0.00000222	393	1384	22	51	0.00000163	695	1384	22	83	0.00000129
	Firefly	293	1348	58	40	0.00000193	406	1348	58	38	0.00000073	721	1348	58	57	0.00000053
DM	GMM	300	1362	44	33	0.00000102	397	1362	44	47	0.00000132	754	1362	44	24	0.00000009
	EM	284	1348	58	49	0.00000350	389	1348	58	56	0.00000213	697	1348	58	81	0.00000125
	BDLC	279	1340	66	54	0.00000439	395	1340	66	49	0.00000162	624	1340	66	154	0.00000740
	Firefly	275	1333	73	58	0.00000536	391	1333	73	53	0.00000205	705	1333	73	73	0.00000109
LE	GMM	289	1333	73	44	0.00000266	402	1333	73	42	0.00000109	746	1333	73	32	0.00000037
	EM	295	1282	124	38	0.00000234	391	1282	124	53	0.00000252	697	1282	124	81	0.00000184
	BDLC	286	1260	146	47	0.00000400	382	1260	146	62	0.00000400	711	1260	146	67	0.00000173
	Firefly	283	1304	102	50	0.00000405	397	1304	102	47	0.00000168	729	1304	102	49	0.00000072

## 7 Hyperparameter tuning approaches for enhancement of classifiers

In order to build a machine learning algorithm with excellent performance, one of the most important steps is to tune the classification model's hyperparameters. In this paper, Grid Search Optimization (GSO) and the Adam technique are used to tune the hyperparameters of several classification algorithms.

### 7.1 Grid Search Optimization (GSO) approach

**Fig. 5:**
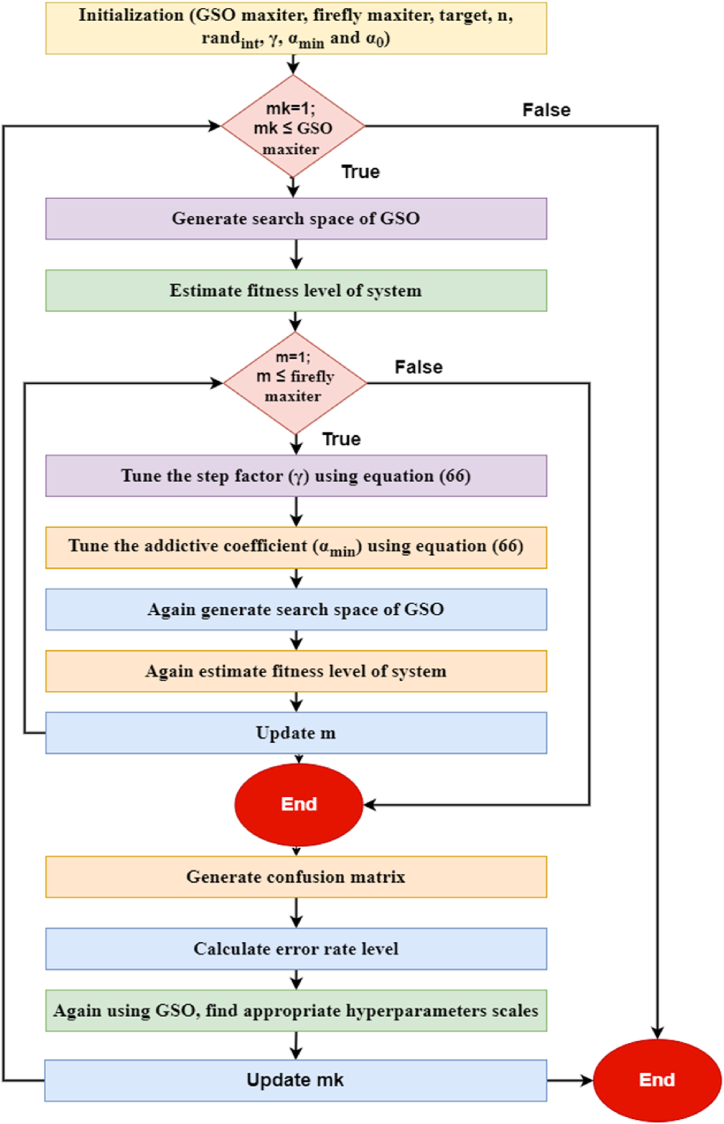
Flowchart of the GSO hyperparameter methodology for the firefly classification algorithm.

In several machine learning strategies, GSO is employed to find the ideal parameters. Cross-validation is taken into consideration in order to direct the outcome metrics [46]. A grid search is an exhaustive search that may be put to use in the process of computing the ideal values for various hyperparameters [47]. It can develop a concept that produces every possible set of parameters and then record each of those combinations. This approach can save time and resources. Once these parameters are tuned, several classifier approaches are obtained [48]. By tweaking hyperparameters, GSO delivers the finest possible solution. Fig. 5 presents the flowchart of the GSO hyperparameter methodology for the firefly classification algorithm. Algorithm 1 in the appendix outlines the strategy for optimizing firefly hyperparameters employing the GSO approach. mk and mk−1 denotes the present and past iterations of the GSO optimizer.

In this work, the firefly classification model hyperparameters are γ, $\alpha_{\min}$ and rand. By tuning the hyperparameters γ, $\alpha_{\min}$ and rand, the effectiveness of the firefly approach can be optimized. The firefly hyperparameter is initialized with a random value within the range [0, 1] using ${\;rand}_{\mathrm{int}}$. The optimal results for firefly maximum iteration (maxiter) and GSO maximum iteration (maxiter) are proved to be 1000 and 500, accordingly. As a result, the firefly classifier's population size (n) is 40, which is taken into account in this study. The hyperparameter values that are most strongly associated with the lowest possible error percentage are identified via this iterative process and recognized as the classiest hyperparameters. The GSO hyperparameter optimization process will also be used with the GMM, EM, and BDLC classifiers to optimize hyperparameters similarly.

### 7.2 Adaptive moment estimation (Adam) approach

Stochastic optimization is an essential part of both deep neural networks and machine learning approaches, and Adam provides a way to carry out this process [49]. The Adam method is easy to develop, fast, and memory-friendly, making it ideal for instances where vast datasets and factors are involved. The adaptive gradients and RMS propagation methods of stochastic gradient descent are both included in the Adam procedure [50]. This optimization technique employs a randomly chosen data segment to build a stochastic approximation, as opposed to utilizing the complete dataset to compute the original gradient. This enables the system to provide a more accurate result. Adam exploits exponential moving and squared gradient approximations. The following expressions evaluate hyperparameters [51]:

(68) $$g_{t+1}=g_{t}-\frac{l}{\sqrt{{\hat{K}}_{t}}+\in }\times {\hat{D}}_{t}$$

(69) $${\hat{D}}_{t}=\frac{D_{t}}{1-R_{1}^{t}}$$

(70) $${\hat{K}}_{t}=\frac{K_{t}}{1-R_{2}^{t}}$$

(71) $$D_{t}=R_{1}D_{t-1}+\left(1-R_{1}\right)\times \frac{\partial l}{\partial g_{t}}$$

(72) $$K_{t}=R_{2}K_{t-1}+\left(1-R_{2}\right)\times {\left(\frac{\partial l}{\partial g_{t}}\right)}^{2}$$

Where ${\hat{D}}_{t}$ represents the first moment estimation, ${\hat{K}}_{t}$ represents the second moment estimation, $g_{t}$ represents the ancient hyperparameters, $g_{t+1}$ represents the tuned hyperparameters, l represents the learning rate of the gradients, ∈, $R_{1}$ and $R_{2}$ represents the constants and $\frac{\partial l}{\partial g_{t}}$ represents the loss function of the gradient to be curtailed at g. As a result, the loss function of the gradient is written mathematically as follows:

(73) $$\frac{\partial l}{\partial g_{pq}}=\frac{e_{pq}}{g_{ini}}\;if\;pq=1$$

(74) $$\frac{\partial l}{\partial g_{pq}}=\frac{e_{pq}-e_{pq-1}}{g_{pq}-g_{pq-1}}\;if\;pq>1$$

**Fig. 6:**
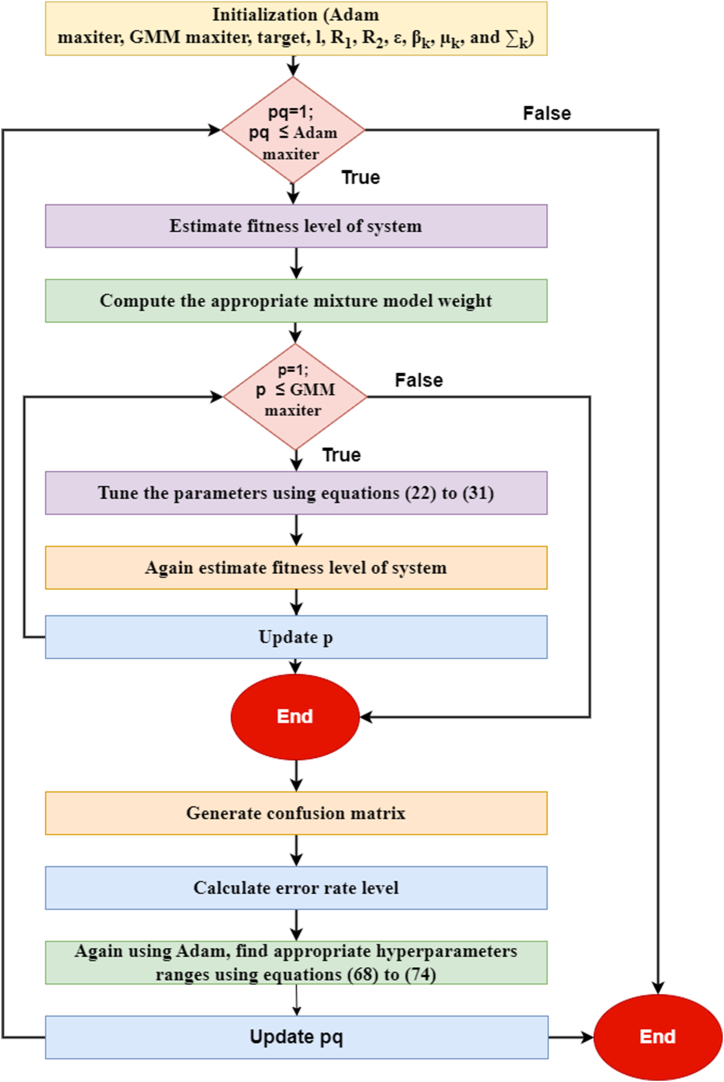
Flowchart of the Adam hyperparameters methodology for the GMM classification algorithm.

Where pq and pq−1 denote the present and past iterations of the Adam optimizer and e represent the error rate of the model. The flowchart of the Adam hyperparameter methodology for the GMM classification algorithm is shown in Fig. 6. Algorithm 2 in the appendix outlines the strategy for optimizing GMM hyperparameters employing the Adam approach. The rate of errors is a loss function that has to be reduced as much as possible. In the GMM model, the hyperparameters $\beta_{k}$, $\mu_{k}$ and $\sum_{k}$ will be used rather than the hyperparameter g, which was employed in the equations shown previously. In this study, the values l=0.0009, $R_{1}=0.74$, $R_{2}$ = 0.82, and $\in =10^{-7}$ have been assigned to the Adam constants. The optimal results for GMM maximum iteration (maxiter) and Adam maximum iteration (maxiter) are proved to be 750 and 300, accordingly. The hyperparameter values that are most strongly associated with the lowest possible error percentage are identified via this iterative process and recognized as sophisticated hyperparameters. The Adam hyperparameter optimization process will also be used with the EM, BDLC and firefly classifiers to optimize hyperparameters similarly. Table 9 provides an analysis of the hyperparameters of the different classifiers in addition to the limiting values for each.

**Table 9:** Different classifiers' hyperparameters and their ranges.

Classifiers	Hyperparameters	Selection Limit		Finest Range	
		Lower	Upper	GSO	Adam
GMM	$\beta_{k}$	0	1	0.826	0.412
	$\mu_{k}$	0	1	0.867	0.482
	$\sum_{k}$	0	1	0.913	1.23E-07
EM	K	0	1	0.981	0.781
BDLC	$\mu_{m}$	0	1	0.965	0.721
	∈	0	1	0.934	0.754
Firefly	γ	0	1	0.801	0.603
	$\alpha_{\min}$	0	1	0.789	0.587
	rand	0	1	0.792	0.554
	$\alpha_{0}$	0	1	0.812	0.623

## 8 Results and discussion

The performance metrics are evaluated in this work. Metrics such as OA, F1 Score, GDR, MCC, and ER are valued from the confusion matrix [44,52]. The True Positive (TP), True Negative (TN), False Positive (FP), and False Negative (FN) are the four parameters that comprise the confusion matrix. The number of accurately identified abnormal segments is represented by TP, the number of accurately identified normal segments is represented by TN, and the number of normal segments that were incorrectly identified is indicated by FP. FN shows the number of incorrectly identified abnormal segments. The mathematical formulae of performance parameter metrics are as follows [53]:

The overall accuracy, which determines the classification system's overall performance, is expressed as follows,

(75) $$OA=\frac{TN+TP}{TN+TP+FN+FP}*100$$

The Error Rate (ER), also known as the misclassification rate, measures the number of samples that have been misclassified into both positive and negative categories, and it is expressed as follows,

(76) $$ER=\frac{FN+FP}{TN+TP+FN+FP}*100$$

Good Detection Ratio (GDR) is mathematically expressed and is a crucial criterion of a detector.

(77) $$GDR=\left(\frac{\left(TP+TN\right)-FP}{\left(TP+TN\right)+FN}\right)*100$$

The F1 Score is the cumulative average of Sensitivity and Specificity, which is determined as follows,

(78) $$F1\;Score=\frac{2TP}{2TP+FP+FN}*100$$

The Matthews Correlation Coefficient (MCC) examines the relationship between the observed and predicted class's classification, and it is expressed as follows [52],

(79) $$MCC=\frac{TP*TN-FP*FN}{\sqrt{\left(TP+FP\right)\left(TP+FN\right)\left(TN+FP\right)\left(TN+FN\right)}}$$

MCC has a value from 0to1. In this work, 0to0.4 values indicate the wrong agreement between the observed and predicted classes of the classifier, and 0.5to1 values represent the perfect agreement between the observed and predicted classes of the classifier. The summarized average result evaluation for VT vs. NSR, PVC vs. NSR, and ST vs. NSR classes of different dimensionally reduced values without CS and HSO feature selection using different classifiers are shown in Table 10. The summarized average result evaluation for VT vs. NSR, PVC vs. NSR, and ST vs. NSR cases of different dimensionally reduced values with CS feature selection using different classifiers are shown in Table 11. The summarized average result evaluation for VT vs. NSR, PVC vs. NSR, and ST vs. NSR classes of different dimensionally reduced values with HSO feature selection using different classifiers are shown in Table 12. Summarized average result analysis for VT vs. NSR, PVC vs. NSR, and ST vs. NSR cases of different dimensionally reduced values with CS feature selection using GSO and Adam hyperparameter tuning based on different classifiers are exposed in Table 13. Summarized average result analysis for VT vs. NSR, PVC vs. NSR, and ST vs. NSR cases of different dimensionally reduced values with HSO feature selection using GSO and Adam hyperparameter tuning based on different classifiers are exposed in Table 14.

**Table 10:** Summarized average result analysis for VT vs. NSR, PVC vs. NSR, and ST vs. NSR classes of different dimensionally reduced values without CS and HSO feature selection using different classifiers.

DR Techniques	Classifiers	VT vs. NSR					PVC vs. NSR					ST vs. NSR				
		OA (%)	F1 Score (%)	GDR (%)	MCC	ER (%)	OA (%)	F1 Score (%)	GDR (%)	MCC	ER (%)	OA (%)	F1 Score (%)	GDR (%)	MCC	ER (%)
LLE	GMM	**57.86**	**39.54**	**37.57**	**0.1394**	**42.14**	**57.93**	**44.76**	**39.77**	**0.1491**	**42.07**	**62.59**	**58.58**	**49.00**	**0.2775**	**37.41**
	EM	54.19	37.15	28.49	0.0923	45.81	54.90	43.26	32.36	0.1130	45.10	59.40	56.21	42.44	0.2250	40.60
	NLR	54.28	35.90	29.55	0.0750	45.72	54.77	41.87	32.92	0.0945	45.23	54.26	46.96	35.77	0.0830	45.74
	LR	55.21	35.69	32.33	0.0759	44.79	57.78	46.16	38.49	0.1665	42.22	55.71	48.24	38.73	0.1095	44.29
	BDLC	53.03	34.14	26.93	0.0454	46.97	53.96	41.10	31.14	0.0800	46.04	57.53	53.09	39.94	0.1738	42.47
	Detrended FA	53.48	34.03	28.31	0.0459	46.52	54.80	41.94	32.98	0.0956	45.20	53.76	46.07	35.07	0.0700	46.24
	Firefly	56.74	38.28	35.19	0.1177	43.26	56.14	42.10	36.58	0.1059	43.86	60.67	56.11	45.92	0.2337	39.33
DM	GMM	54.99	36.26	31.38	0.0828	45.01	54.70	40.73	33.46	0.0802	45.30	56.68	50.50	39.66	0.1398	43.32
	EM	54.57	35.65	30.52	0.0727	45.43	54.51	40.63	33.00	0.0778	45.49	60.95	56.50	46.35	0.2404	39.05
	NLR	54.20	34.60	30.06	0.0568	45.80	54.91	41.31	33.68	0.0886	45.09	57.47	51.92	40.68	0.1611	42.53
	LR	53.93	34.25	29.49	0.0509	46.07	53.66	38.93	31.71	0.0522	46.34	54.38	46.72	36.27	0.0822	45.62
	BDLC	53.92	34.69	29.21	0.0568	46.08	54.03	40.07	32.02	0.0681	45.97	57.43	52.21	40.36	0.1637	42.57
	Detrended FA	51.00	32.77	21.57	0.0169	49.00	53.28	42.18	28.33	0.0895	46.72	51.77	45.03	30.34	0.0394	48.23
	Firefly	**56.09**	**36.61**	**34.28**	**0.0921**	**43.91**	**56.78**	**43.32**	**37.56**	**0.1245**	**43.22**	**61.09**	**57.82**	**45.67**	**0.2574**	**38.91**
LE	GMM	53.47	34.59	27.95	0.0534	46.53	55.08	42.93	33.10	0.1097	44.92	57.49	52.77	40.10	0.1700	42.51
	EM	**55.98**	**35.62**	**34.51**	**0.0783**	**44.02**	**56.70**	**42.59**	**37.77**	**0.1152**	**43.30**	58.40	51.94	43.10	0.1703	41.60
	NLR	55.48	35.03	33.45	0.0683	44.52	55.90	41.38	36.35	0.0958	44.10	56.19	48.39	39.87	0.1159	43.81
	LR	51.68	33.62	23.13	0.0319	48.32	52.15	39.42	27.00	0.0477	47.85	56.61	52.81	37.61	0.1625	43.39
	BDLC	53.96	34.38	29.52	0.0527	46.04	55.16	42.08	33.89	0.0995	44.84	57.81	52.64	41.04	0.1716	42.19
	Detrended FA	53.62	33.99	28.75	0.0460	46.38	53.43	38.81	31.13	0.0493	46.57	54.62	47.39	36.42	0.0905	45.38
	Firefly	55.92	35.48	34.43	0.0762	44.08	56.47	42.11	37.45	0.1081	43.53	**60.19**	**54.94**	**45.56**	**0.2169**	**39.81**

**Table 11:** Summarized average result analysis for VT vs. NSR, PVC vs. NSR, and ST vs. NSR classes of different dimensionally reduced values with CS feature selection using different classifiers.

DR Techniques	Classifiers	VT vs. NSR					PVC vs. NSR					ST vs. NSR				
		OA (%)	F1 Score (%)	GDR (%)	MCC	ER (%)	OA (%)	F1 Score (%)	GDR (%)	MCC	ER (%)	OA (%)	F1 Score (%)	GDR (%)	MCC	ER (%)
LLE	GMM	74.22	52.44	67.43	0.3994	25.78	71.03	50.24	63.97	0.3165	28.97	73.66	66.28	68.42	0.4541	26.34
	EM	68.60	43.66	58.45	0.2723	31.40	69.05	50.84	59.83	0.3195	30.95	69.79	62.21	62.50	0.3816	30.21
	NLR	64.51	40.93	50.28	0.2304	35.49	65.40	48.53	52.66	0.2807	34.60	65.97	58.90	55.92	0.3170	34.03
	LR	65.36	42.14	51.82	0.2492	34.64	63.24	42.63	50.04	0.1930	36.76	63.52	54.13	53.00	0.2476	36.48
	BDLC	57.33	33.60	35.04	0.1102	42.67	58.65	41.89	38.96	0.1683	41.35	58.95	51.61	43.51	0.1822	41.05
	Detrended FA	58.24	35.51	36.63	0.1410	41.76	57.63	39.25	37.64	0.1273	42.37	58.61	50.82	43.17	0.1710	41.39
	Firefly	**76.74**	**53.47**	**71.82**	**0.4105**	**23.26**	**76.39**	**58.75**	**71.75**	**0.4392**	**23.61**	**78.39**	**72.10**	**74.90**	**0.5512**	**21.61**
DM	GMM	68.44	46.09	57.41	0.3093	31.56	66.90	47.96	56.25	0.2752	33.10	67.34	59.09	58.85	0.3295	32.66
	EM	64.91	40.95	51.20	0.2309	35.09	64.83	46.45	52.20	0.2493	35.17	65.47	57.62	55.51	0.2991	34.53
	NLR	64.86	38.36	51.91	0.1930	35.14	66.50	48.56	55.15	0.2828	33.50	67.15	59.58	58.18	0.3332	32.85
	LR	57.36	33.90	34.98	0.1148	42.64	59.46	43.82	40.06	0.1992	40.54	58.21	50.42	42.44	0.1635	41.79
	BDLC	59.79	39.16	39.08	0.2012	40.21	59.70	43.72	40.77	0.1983	40.30	60.31	53.69	45.58	0.2159	39.69
	Detrended FA	59.08	35.97	38.69	0.1495	40.92	58.98	41.00	40.33	0.1564	41.02	59.14	50.98	44.42	0.1774	40.86
	Firefly	**72.58**	**50.23**	**64.80**	**0.3684**	**27.42**	**71.97**	**54.44**	**64.62**	**0.3744**	**28.03**	**70.99**	**62.53**	**64.79**	**0.3944**	**29.01**
LE	GMM	62.21	41.43	44.43	0.2388	37.79	60.83	43.21	43.97	0.1934	39.17	60.61	52.43	47.13	0.2049	39.39
	EM	62.18	38.76	45.48	0.1958	37.82	62.20	44.25	46.92	0.2118	37.80	63.74	56.72	52.05	0.2761	36.26
	NLR	61.55	36.44	44.82	0.1603	38.45	62.31	44.01	47.29	0.2088	37.69	63.71	56.40	52.16	0.2722	36.29
	LR	59.02	34.51	39.16	0.1272	40.98	59.01	40.05	40.88	0.1433	40.99	59.55	51.12	45.35	0.1825	40.45
	BDLC	61.79	38.37	44.67	0.1895	38.21	60.56	40.92	44.38	0.1608	39.44	63.67	56.85	51.78	0.2769	36.34
	Detrended FA	61.14	35.98	43.97	0.1528	38.86	60.86	41.10	45.03	0.1643	39.14	61.88	53.46	49.58	0.2264	38.12
	Firefly	**68.79**	**41.34**	**59.44**	**0.2400**	**31.21**	**68.14**	**46.44**	**59.33**	**0.2592**	**31.86**	**71.90**	**64.82**	**65.58**	**0.4256**	**28.10**

**Table 12:** Summarized average result analysis for VT vs. NSR, PVC vs. NSR, and ST vs. NSR classes of different dimensionally reduced values with HSO feature selection using different classifiers.

DR Techniques	Classifiers	VT vs. NSR					PVC vs. NSR					ST vs. NSR				
		OA (%)	F1 Score (%)	GDR (%)	MCC	ER (%)	OA (%)	F1 Score (%)	GDR (%)	MCC	ER (%)	OA (%)	F1 Score (%)	GDR (%)	MCC	ER (%)
LLE	GMM	**86.40**	**72.30**	**84.50**	**0.6666**	**13.60**	**87.65**	**78.93**	**86.05**	**0.7296**	**12.35**	**89.54**	**86.96**	**88.41**	**0.7979**	**10.46**
	EM	75.89	56.85	69.54	0.4666	24.11	78.66	67.59	73.46	0.5794	21.34	82.11	79.32	78.55	0.6761	17.89
	NLR	76.01	52.51	70.71	0.3975	23.99	77.79	62.89	73.24	0.4987	22.21	72.41	61.94	67.76	0.4033	27.59
	LR	62.96	41.73	46.19	0.2434	37.04	62.52	45.76	47.00	0.2347	37.48	68.42	64.63	58.00	0.4077	31.58
	BDLC	69.59	46.41	59.77	0.3131	30.41	69.05	50.84	59.83	0.3195	30.95	69.14	61.08	61.69	0.3646	30.86
	Detrended FA	69.22	42.59	59.99	0.2577	30.78	70.64	52.63	62.52	0.3471	29.36	70.41	62.24	63.75	0.3866	29.59
	Firefly	77.29	55.06	72.48	0.4328	22.71	80.01	67.16	76.09	0.5626	19.99	75.33	66.83	71.34	0.4735	24.67
DM	GMM	**81.78**	**65.58**	**78.21**	**0.5850**	**18.22**	83.82	74.18	80.86	0.6714	16.18	85.81	82.95	83.68	0.7342	14.19
	EM	77.30	51.01	73.18	0.3777	22.70	**84.05**	**73.83**	**81.36**	**0.6613**	**15.95**	**86.28**	**83.27**	**84.36**	**0.7377**	**13.72**
	NLR	77.46	51.08	73.43	0.3788	22.54	78.95	62.05	75.46	0.4868	21.05	78.75	71.33	75.83	0.5459	21.25
	LR	70.91	45.55	62.66	0.3001	29.09	71.03	52.19	63.43	0.3417	28.97	70.99	62.53	64.79	0.3944	29.01
	BDLC	78.04	60.12	72.76	0.5126	21.96	73.45	54.12	67.54	0.3725	26.55	73.03	64.09	68.11	0.4273	26.97
	Detrended FA	76.24	53.94	70.83	0.4181	23.76	73.49	52.93	67.90	0.3579	26.51	72.49	62.45	67.73	0.4081	27.51
	Firefly	81.63	64.04	78.24	0.5583	18.37	79.61	64.15	76.11	0.5161	20.40	80.36	74.30	77.58	0.5887	19.64
LE	GMM	**79.17**	**59.28**	**74.95**	**0.4921**	**20.83**	**82.67**	**72.20**	**79.41**	**0.6405**	**17.33**	**85.11**	**82.09**	**82.80**	**0.7192**	**14.89**
	EM	78.20	58.65	73.39	0.4861	21.80	82.11	72.11	78.44	0.6444	17.89	84.66	81.89	82.07	0.7185	15.34
	NLR	77.88	58.45	72.85	0.4842	22.12	80.02	68.22	75.81	0.5819	19.98	71.33	60.23	66.37	0.3783	28.67
	LR	66.28	43.57	53.44	0.2712	33.72	67.20	51.08	55.72	0.3212	32.80	66.17	58.41	56.65	0.3132	33.83
	BDLC	71.16	48.10	62.52	0.3375	28.84	69.23	49.21	60.72	0.2979	30.77	69.49	60.61	62.63	0.3628	30.51
	Detrended FA	66.64	44.00	54.10	0.2779	33.36	65.32	46.38	53.33	0.2496	34.68	68.09	61.48	59.22	0.3616	31.91
	Firefly	70.68	48.35	61.47	0.3421	29.32	71.35	55.43	63.05	0.3889	28.65	70.21	62.54	63.21	0.3883	29.79

**Table 13:** Summarized average result analysis for VT vs. NSR, PVC vs. NSR, and ST vs. NSR classes of different dimensionally reduced values with CS feature selection using different classifiers for GSO and Adam hyperparameter tuning.

CS features with GSO hyperparameter tuning																
DR Techniques	Classifiers	VT vs. NSR					PVC vs. NSR					ST vs. NSR				
		OA (%)	F1 Score (%)	GDR (%)	MCC	ER (%)	OA (%)	F1 Score (%)	GDR (%)	MCC	ER (%)	OA (%)	F1 Score (%)	GDR (%)	MCC	ER (%)
LLE	GMM	84.20	66.33	82.00	0.5812	15.80	83.34	69.31	81.18	0.5882	16.66	84.99	80.17	83.38	0.6847	15.01
	EM	81.27	61.82	78.04	0.5238	18.73	79.35	62.49	76.03	0.4934	20.65	77.50	68.85	74.51	0.5126	22.50
	BDLC	80.94	61.86	77.48	0.5259	19.06	77.86	59.58	74.13	0.4530	22.14	77.60	69.46	74.48	0.5185	22.40
	Firefly	**86.47**	**70.74**	**84.84**	**0.6387**	**13.53**	**86.35**	**74.96**	**84.81**	**0.6666**	**13.65**	**87.18**	**83.03**	**85.99**	**0.7309**	**12.82**
DM	GMM	79.80	56.38	76.48	0.4487	20.20	79.95	63.18	76.88	0.5033	20.05	79.59	72.15	77.00	0.5611	20.41
	EM	79.54	57.89	75.83	0.4699	20.46	80.05	64.97	76.68	0.5277	19.95	76.35	67.19	73.05	0.4871	23.65
	BDLC	76.06	51.43	70.99	0.3824	23.94	80.57	68.38	76.76	0.5815	19.43	74.54	65.40	70.42	0.4535	25.46
	Firefly	**84.11**	**66.65**	**81.81**	**0.5868**	**15.89**	**82.75**	**68.35**	**80.43**	**0.5750**	**17.25**	**82.89**	**76.96**	**80.98**	**0.6353**	**17.11**
LE	GMM	76.47	53.85	71.27	0.4163	23.53	76.11	58.91	71.21	0.4410	23.89	75.84	68.18	71.77	0.4907	24.16
	EM	74.22	47.99	68.35	0.3352	25.78	75.49	57.98	70.32	0.4275	24.51	75.04	67.07	70.72	0.4732	24.96
	BDLC	77.25	50.85	73.12	0.3756	22.75	78.30	60.69	74.64	0.4681	21.70	77.67	69.58	74.56	0.5203	22.33
	Firefly	**81.91**	**62.63**	**78.94**	**0.5338**	**18.09**	**82.56**	**69.43**	**79.89**	**0.5911**	**17.44**	**82.84**	**77.57**	**80.66**	**0.6418**	**17.16**

CS features with Adam hyperparameter tuning																
DR Techniques	Classifiers	VT vs. NSR					PVC vs. NSR					ST vs. NSR				
		OA (%)	F1 Score (%)	GDR (%)	MCC	ER (%)	OA (%)	F1 Score (%)	GDR (%)	MCC	ER (%)	OA (%)	F1 Score (%)	GDR (%)	MCC	ER (%)
LLE	GMM	**91.71**	**80.17**	**91.19**	**0.7540**	**8.29**	**91.59**	**83.39**	**91.09**	**0.7797**	**8.42**	**92.25**	**89.36**	**91.87**	**0.8332**	**7.75**
	EM	89.20	74.18	88.37	0.6780	10.80	90.09	80.77	89.37	0.7445	9.91	90.59	87.16	90.03	0.7982	9.41
	BDLC	83.00	62.83	80.64	0.5336	17.00	84.27	71.53	82.24	0.6193	15.73	82.58	76.22	80.70	0.6256	17.42
	Firefly	87.17	70.27	85.93	0.6287	12.83	87.94	77.06	86.85	0.6944	12.06	86.28	80.84	85.24	0.7016	13.72
DM	GMM	**86.40**	**69.73**	**84.87**	**0.6232**	**13.60**	**87.50**	**77.06**	**86.19**	**0.6957**	**12.50**	**86.67**	**81.98**	**85.50**	**0.7155**	**13.33**
	EM	84.12	64.15	82.16	0.5502	15.88	85.84	73.97	84.21	0.6528	14.16	85.90	81.10	84.56	0.7008	14.10
	BDLC	81.11	59.23	78.17	0.4865	18.89	82.59	68.86	80.06	0.5823	17.41	81.11	74.41	78.84	0.5956	18.89
	Firefly	83.92	64.90	81.78	0.5609	16.08	84.67	72.04	82.76	0.6262	15.33	84.54	79.26	82.94	0.6715	15.46
LE	GMM	**85.67**	**68.47**	**83.95**	**0.6075**	**14.33**	**86.16**	**74.53**	**84.60**	**0.6605**	**13.84**	**86.26**	**81.58**	**84.97**	**0.7084**	**13.74**
	EM	84.87	67.64	82.85	0.5984	15.13	84.92	72.62	83.04	0.6345	15.08	85.05	80.08	83.50	0.6839	14.95
	BDLC	81.70	62.36	78.64	0.5305	18.30	81.98	68.33	79.18	0.5753	18.02	81.83	76.05	79.49	0.6180	18.17
	Firefly	84.42	66.35	82.34	0.5807	15.58	84.54	71.75	82.62	0.6221	15.46	84.31	78.89	82.68	0.6658	15.69

**Table 14:** Summarized average result analysis for VT vs. NSR, PVC vs. NSR, and ST vs. NSR classes of different dimensionally reduced values with HSO feature selection using different classifiers for Adam hyperparameter tuning.

HSO features with GSO hyperparameter tuning																
DR Techniques	Classifiers	VT vs. NSR					PVC vs. NSR					ST vs. NSR				
		OA (%)	F1 Score (%)	GDR (%)	MCC	ER (%)	OA (%)	F1 Score (%)	GDR (%)	MCC	ER (%)	OA (%)	F1 Score (%)	GDR (%)	MCC	ER (%)
LLE	GMM	90.01	77.45	89.14	0.7228	9.99	90.42	82.08	89.64	0.7638	9.58	89.92	86.37	89.24	0.7852	10.08
	EM	87.50	72.83	86.09	0.6658	12.50	86.00	73.58	84.53	0.6471	14.00	83.79	77.21	82.37	0.6463	16.21
	BDLC	86.96	72.34	85.36	0.6619	13.04	84.34	70.40	82.54	0.6035	15.66	84.23	78.38	82.73	0.6602	15.77
	Firefly	**93.75**	**85.17**	**93.42**	**0.8180**	**6.25**	**93.75**	**87.80**	**93.44**	**0.8393**	**6.25**	**93.38**	**90.89**	**93.10**	**0.8573**	**6.62**
DM	GMM	87.69	71.27	86.56	0.6413	12.31	87.71	76.16	86.66	0.6823	12.29	87.92	83.35	87.05	0.7391	12.08
	EM	85.90	68.27	84.32	0.6037	14.10	86.75	75.35	85.36	0.6715	13.25	85.18	79.57	83.88	0.6797	14.82
	BDLC	84.04	64.61	82.00	0.5565	15.96	86.57	75.98	84.96	0.6823	13.43	83.71	77.71	82.09	0.6494	16.29
	Firefly	**90.95**	**79.23**	**90.25**	**0.7443**	**9.05**	**91.15**	**83.18**	**90.51**	**0.7778**	**8.85**	**90.22**	**86.58**	**89.63**	**0.7894**	**9.78**
LE	GMM	84.57	66.78	82.51	0.5866	15.43	84.67	72.04	82.76	0.6262	15.33	83.56	77.66	81.86	0.6476	16.44
	EM	81.59	60.70	78.73	0.5062	18.41	82.78	69.30	80.28	0.5885	17.22	82.60	76.88	80.50	0.6323	17.40
	BDLC	84.34	63.99	82.52	0.5480	15.66	84.70	70.75	83.04	0.6087	15.30	83.72	77.26	82.25	0.6459	16.28
	Firefly	**88.22**	**74.33**	**86.96**	**0.6853**	**11.78**	**87.52**	**76.78**	**86.27**	**0.6911**	**12.48**	**87.28**	**82.80**	**86.22**	**0.7285**	**12.72**

HSO features with Adam hyperparameter tuning																
DR Techniques	Classifiers	VT vs. NSR					PVC vs. NSR					ST vs. NSR				
		OA (%)	F1 Score (%)	GDR (%)	MCC	ER (%)	OA (%)	F1 Score (%)	GDR (%)	MCC	ER (%)	OA (%)	F1 Score (%)	GDR (%)	MCC	ER (%)
LLE	GMM	**98.38**	**95.69**	**98.38**	**0.9473**	**1.62**	**98.48**	**96.79**	**98.47**	**0.9581**	**1.52**	**98.92**	**98.48**	**98.92**	**0.9765**	**1.08**
	EM	95.48	88.37	95.37	0.8558	4.52	95.88	91.52	95.78	0.8882	4.12	94.93	92.78	94.83	0.8890	5.07
	BDLC	95.90	88.83	95.84	0.8644	4.10	96.07	91.53	96.02	0.8905	3.93	95.19	92.98	95.15	0.8951	4.81
	Firefly	94.34	85.63	94.15	0.8216	5.66	94.78	89.36	94.61	0.8594	5.22	94.73	92.60	94.58	0.8851	5.27
DM	GMM	**95.58**	**88.65**	**95.47**	**0.8593**	**4.42**	**95.07**	**89.68**	**94.95**	**0.8644**	**4.93**	**96.88**	**95.68**	**96.82**	**0.9325**	**3.12**
	EM	93.79	84.02	93.58	0.8018	6.21	93.84	87.21	93.64	0.8315	6.16	93.61	90.90	93.44	0.8601	6.39
	BDLC	93.12	82.36	92.85	0.7811	6.88	93.82	87.37	93.59	0.8330	6.18	89.94	85.03	89.62	0.7781	10.06
	Firefly	92.45	80.73	92.12	0.7606	7.55	93.17	86.08	92.89	0.8160	6.83	93.31	90.61	93.08	0.8542	6.69
LE	GMM	**93.25**	**83.11**	**92.95**	**0.7900**	**6.75**	**93.80**	**87.52**	**93.54**	**0.8348**	**6.20**	**95.17**	**93.39**	**95.00**	**0.8965**	**4.83**
	EM	90.65	78.39	89.93	0.7331	9.35	90.40	81.48	89.71	0.7543	9.60	90.59	87.16	90.03	0.7982	9.41
	BDLC	88.89	74.77	87.87	0.6875	11.11	88.71	78.52	87.74	0.7143	11.29	90.24	86.96	89.54	0.7943	9.76
	Firefly	91.21	78.73	90.66	0.7354	8.79	91.90	84.11	91.42	0.7894	8.10	93.08	90.61	92.74	0.8526	6.92

Table 10 represents the consolidated average result evaluation for VT vs. NSR, PVC vs. NSR, and ST vs. NSR classes of different dimensionally reduced values without CS and HSO feature selection using different classifiers. Since the dimensionality reduction was carried out using the LLE technique without feature selection, the output of the classifier with an error rate range attained from 42.14 % to 46.97 % in the VT vs. NSR Case. The GMM Classifier attains high parametric average values, such as 57.86 % overall accuracy, 39.54 % F1 score, 37.57 % GDR, and 0.1394 MCC. In the PVC vs. NSR Case, the error rate range attained from 42.07 % to 46.04 %. The GMM Classifier attains high parametric average values, such as 57.93 % overall accuracy, 44.76 % F1 score, 39.77 % GDR, and 0.1491 MCC. In the ST vs. NSR case, the error rate ranged from 37.41 % to 46.24 %. The GMM Classifier attains high parametric average values, such as 62.59 % overall accuracy, 58.58 % F1 score, 49.00 % GDR, and 0.2775 MCC. Since the dimensionality reduction was carried out using the DM technique without feature selection, the output of the classifier with an error rate range attained from 43.91 % to 49 % in the VT vs. NSR Case. The Firefly classifier attains high parametric average values, such as 56.09 % overall accuracy, 36.61 % F1 score, 34.28 % GDR, and 0.0921MCC. In the PVC vs. NSR Case, the error rate range attained from 43.22 % to 46.72 %. The Firefly classifier attains high parametric average values, such as 56.78 % overall accuracy, 43.32 % F1 score, 37.56 % GDR, and 0.1245 MCC. In the ST vs. NSR case, the error rate ranged from 38.91 % to 48.23 %. The Firefly classifier attains high parametric average values, such as 61.09 % overall accuracy, 57.82 % F1 score, 45.67 % GDR, and 0.2574 MCC. Since the dimensionality reduction was carried out using the LE technique without feature selection and the output of the classifier with an error rate range attained from 44.02 % to 48.32 % in the VT vs. NSR Case. The EM Classifier attains high parametric average values, such as 55.98 % overall accuracy, 35.62 % F1 score, 34.51 % GDR, and 0.0783 MCC. In the PVC vs. NSR Case, the error rate range attained from 43.3 % to 47.85 %. The EM classifier attains high parametric average values, such as 56.70 % overall accuracy, 42.59 % F1 score, 37.77 % GDR, and 0.1152 MCC. In the ST vs. NSR case, the error rate ranged from 39.81 % to 45.38 %. The Firefly classifier attains high parametric average values, such as 60.19 % overall accuracy, 54.94 % F1 score, 45.56 % GDR, and 0.2169 MCC.

Table 11 exhibits the consolidated average result evaluation for VT vs. NSR, PVC vs. NSR, and ST vs. NSR classes of different dimensionally reduced values with CS feature selection using different classifiers. Since the dimensionality reduction was carried out using the LLE technique with CS feature selection and the output of the classifier with error rate range attained from 23.26 % to 42.67 % in VT vs. NSR Case. The Firefly classifier attains high parametric average values, such as 76.74 % overall accuracy, 53.47 % F1 score, 71.82 % GDR, and 0.4105 MCC. In the PVC vs. NSR Case, the error rate range attained from 23.61 % to 42.37 %. The Firefly classifier attains high parametric average values, such as 76.39 % overall accuracy, 58.75 % F1 score, 71.75 % GDR, and 0.4392 MCC. In the ST vs. NSR case, the error rate ranged from 21.61 % to 41.39 %. The Firefly classifier attains high parametric average values, such as 78.39 % overall accuracy, 72.10 % F1 score, 74.90 % GDR, and 0.5512 MCC. Since the dimensionality reduction was carried out using the DM technique with CS feature selection and the output of the classifier with error rate range attained from 27.42 % to 42.64 % in VT vs. NSR Case. The Firefly classifier attains high parametric average values, such as 72.58 % overall accuracy, 50.23 % F1 score, 64.80 % GDR, and 0.3684 MCC. In the PVC vs. NSR Case, the error rate range attained from 28.03 % to 41.02 %. The Firefly classifier attains high parametric average values, such as 71.97 % overall accuracy, 54.44 % F1 score, 64.62 % GDR, and 0.3744 MCC. In the ST vs. NSR case, the error rate ranged from 29.01 % to 41.79 %. The Firefly classifier attains high parametric average values, such as 70.99 % overall accuracy, 62.53 % F1 score, 64.79 % GDR, and 0.3944 MCC. Since the dimensionality reduction was carried out using the LE technique with CS feature selection and the output of the classifier with error rate range attained from 31.21 % to 40.98 % in VT vs. NSR Case. The Firefly classifier attains high parametric average values, such as 68.79 % overall accuracy, 41.34 % F1 score, 59.44 % GDR, and 0.2400 MCC. In the PVC vs. NSR Case, the error rate range attained from 31.86 % to 40.99 %. The Firefly classifier attains high parametric average values, such as 68.14 % overall accuracy, 46.44 % F1 score, 59.33 % GDR, and 0.2592 MCC. In the ST vs. NSR case, the error rate ranged from 28.1 % to 40.45 %. The Firefly Classifier attains high parametric average values, such as 71.90 % overall accuracy, 64.82 % F1 score, 65.58 % GDR, and 0.4256 MCC.

Table 12 exhibits the consolidated average result evaluation for VT vs. NSR, PVC vs. NSR, and ST vs. NSR classes of different dimensionally reduced values with HSO feature selection using different classifiers. Since the dimensionality reduction was carried out using the LLE technique with HSO feature selection and the output of the classifier with error rate range attained from 13.6 % to 37.04 % in VT vs. NSR Case. The GMM classifier attains high parametric average values, such as 86.40 % overall accuracy, 72.30 % F1 score, 84.50 % GDR, and 0.6666 MCC. In the PVC vs. NSR Case, the error rate range attained from 12.35 % to 37.48 %. The GMM classifier attains high parametric average values, such as 87.65 % overall accuracy, 78.93 % F1 score, 86.05 % GDR, and 0.7296 MCC. In the ST vs. NSR case, the error rate ranged from 10.46 % to 31.58 %. The GMM classifier attains high parametric average values, such as 89.54 % overall accuracy, 86.96 % F1 score, 88.41 % GDR, and 0.7979 MCC. Since the dimensionality reduction was carried out using the DM technique with HSO feature selection and the output of the classifier with error rate range attained from 18.22 % to 29.09 % in VT vs. NSR Case. The GMM classifier attains high parametric average values, such as 81.78 % overall accuracy, 65.58 % F1 score, 78.21 % GDR, and 0.5850 MCC. In the PVC vs. NSR Case, the error rate range attained from 15.95 % to 28.97 %. The EM classifier attains high parametric average values, such as 84.05 % overall accuracy, 73.83 % F1 score, 81.36 % GDR, and 0.6613 MCC. In the ST vs. NSR case, the error rate ranged from 13.72 % to 29.01 %. The EM classifier attains high parametric average values, such as 86.28 % overall accuracy, 83.27 % F1 score, 84.36 % GDR, and 0.7377 MCC. Since the dimensionality reduction was carried out using the LE technique with HSO feature selection and the output of the classifier with error rate range attained from 20.83 % to 33.72 % in VT vs. NSR Case. The GMM classifier attains high parametric average values, such as 79.17 % overall accuracy, 59.28 % F1 score, 74.95 % GDR, and 0.4921 MCC. In the PVC vs. NSR Case, the error rate range attained from 17.33 % to 34.68 %. The GMM classifier attains high parametric average values, such as 82.67 % overall accuracy, 72.20 % F1 score, 79.41 % GDR, and 0.6405 MCC. In the ST vs. NSR case, the error rate ranged from 14.89 % to 33.83 %. The GMM Classifier attains high parametric average values, such as 85.11 % overall accuracy, 82.09 % F1 score, 82.80 % GDR, and 0.7192 MCC.

**Fig. 7:**
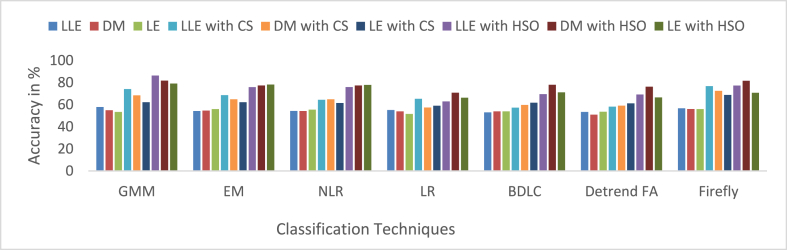
Performance of average accuracy for different dimensionality reduction techniques with and without CS and HSO feature selection of different classifiers for the VT vs. NSR Case.

**Fig. 8:**
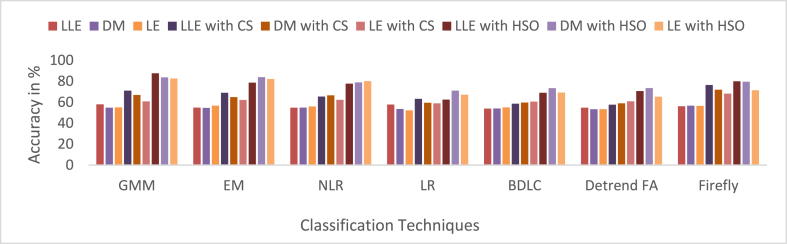
Performance of average accuracy for different dimensionality reduction techniques with and without CS and HSO feature selection of different classifiers for the PVC vs. NSR Case.

**Fig. 9:**
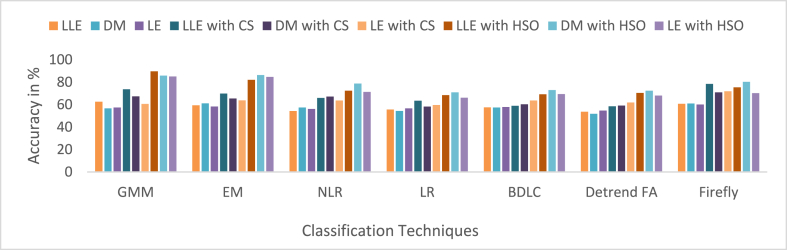
Performance of average accuracy for different dimensionality reduction techniques with and without CS and HSO feature selection of different classifiers for the ST vs. NSR Case.

**Fig. 10:**
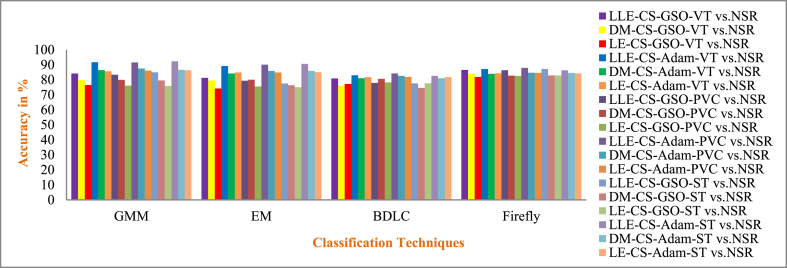
Performance of average accuracy for different dimensionality reduction techniques for CS feature selection with different classifiers based GSO and Adam hyperparameter tuning for VT vs. NSR, PVC vs. NSR and ST vs. NSR cases.

**Fig. 11:**
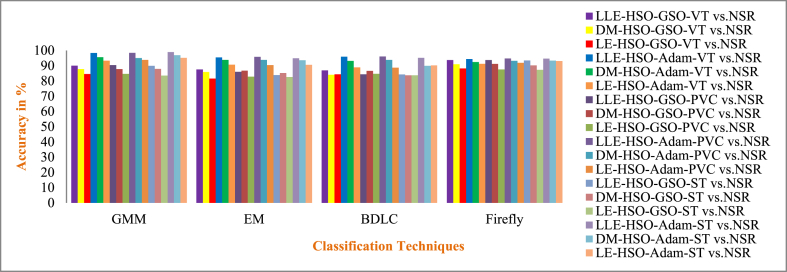
Performance of average accuracy for different dimensionality reduction techniques for HSO feature selection with different classifiers based GSO and Adam hyperparameter tuning for VT vs. NSR, PVC vs. NSR and ST vs. NSR cases.

Table 13, Table 14 indicate the summary of the average result analysis for VT vs. NSR, PVC vs. NSR, and ST vs. NSR classes of different dimensionally reduced values with CS and HSO feature selection using different classifiers for GSO and Adam hyperparameter tuning. The performance of GMM, EM, BDLC, and firefly classifications is substantially enriched when the hyperparameters are optimized by employing the Adam and GSO algorithms. The Adam hyperparameter tuning surpasses all other techniques for identifying the ST vs. NSR class, with an overall accuracy of 98.92 % when implementing the LLE with HSO's features-based GMM classifier. Table 15 expresses the results of calculating the computational complexity of the VT vs. NSR, PVC vs. NSR, and ST vs. NSR cases of different dimensionally reduced values with CS and HSO feature selection using GSO and Adam hyperparameter tuning approaches based on different classifiers. From Tables 15 and it is clear that the HSO feature selection with an Adam hyperparameter tuning-based GMM classification model for LLE dimensionality reduction in ST vs. NSR cases has a higher computational complexity of $O\left({2n}^{8}\;\log\;9\;n\right)$ with a greater execution time of 550 s. Fig. 7 displays the average accuracy performance of different dimensionality reduction techniques with and without CS and HSO feature selection of different classifiers for the VT vs. NSR Case. Fig. 8 exhibits the performance of average accuracy for different dimensionality reduction techniques with and without CS and HSO feature selection of different classifiers for PVC vs. NSR Case. Fig. 9 illustrates the performance of average accuracy for different dimensionality reduction techniques with and without CS and HSO feature selection of different classifiers for ST vs. NSR Case. Fig. 10 illustrates the performance of average accuracy for different dimensionality reduction techniques for CS feature selection with different classifiers based on GSO and Adam hyperparameter tuning for VT vs. NSR, PVC vs. NSR and ST vs. NSR cases. Fig. 11 illustrates the performance of average accuracy for different dimensionality reduction techniques for HSO feature selection with different classifiers based on GSO and Adam hyperparameter tuning for VT vs. NSR, PVC vs. NSR and ST vs. NSR cases. When compared to other classifiers, Fig. 7, Fig. 8, Fig. 9, Fig. 10, Fig. 11 illustrate that the HSO feature selection classifiers with Adam hyperparameter tuning outperform them in terms of overall accuracy.

**Table 15:** Computational complexity for VT vs. NSR, PVC vs. NSR, and ST vs. NSR cases of different dimensionally reduced ideals with CS and HSO feature selection using different classifiers for GSO and Adam hyperparameter tuning approaches.

CVDs	Classification Techniques	DR Approaches	without CS and HSO feature selection	with CS feature selection	with HSO feature selection	with CS feature selection – GSO	with CS feature selection – Adam	with HSO feature selection – GSO	with HSO feature selection – Adam
VT vs. NSR, PVC vs. NSR, and ST vs. NSR	GMM	LLE	O $\left({2n}^{7}\;\log\;n\right)$	O $\left({2n}^{7}\;\log\;2\;n\right)$	O $\left({2n}^{8}\;\log\;2\;n\right)$	O $\left({2n}^{8}\;\log\;3\;n\right)$	O $\left({2n}^{8}\;\log\;5\;n\right)$	O $\left({2n}^{8}\;\log\;7\;n\right)$	O $\left({2n}^{8}\;\log\;9\;n\right)$
		DM	O $\left(4n^{6}\;\log\;n\right)$	O $\left(4n^{6}\;\log\;2\;n\right)$	O $\left(4n^{7}\;\log\;2\;n\right)$	O $\left(4n^{7}\;\log\;3\;n\right)$	O $\left(4n^{7}\;\log\;5\;n\right)$	O $\left(4n^{7}\;\log\;7\;n\right)$	O $\left(4n^{7}\;\log\;9\;n\right)$
		LE	O $\left(4n^{7}\;\log\;n\right)$	O $\left(4n^{7}\;\log\;2\;n\right)$	O $\left(4n^{8}\;\log\;2\;n\right)$	O $\left(4n^{8}\;\log\;3\;n\right)$	O $\left(4n^{8}\;\log\;5\;n\right)$	O $\left(4n^{8}\;\log\;7\;n\right)$	O $\left(4n^{8}\;\log\;9\;n\right)$
	EM	LLE	O $\left({2n}^{4}\;\log\;n\right)$	O $\left({2n}^{4}\;\log\;2n\right)$	O $\left({2n}^{5}\;\log\;2n\right)$	O $\left({2n}^{5}\;\log\;3n\right)$	O $\left({2n}^{5}\;\log\;5n\right)$	O $\left({2n}^{5}\;\log\;7n\right)$	O $\left({2n}^{5}\;\log\;9n\right)$
		DM	O $\left(4n^{3}\;\log\;n\right)$	O $\left(4n^{3}\;\log\;2\;n\right)$	O $\left(4n^{4}\;\log\;2\;n\right)$	O $\left(4n^{4}\;\log\;3\;n\right)$	O $\left(4n^{4}\;\log\;5\;n\right)$	O $\left(4n^{4}\;\log\;7\;n\right)$	O $\left(4n^{4}\;\log\;9\;n\right)$
		LE	O $\left(4n^{4}\;\log\;n\right)$	O $\left(4n^{4}\;\log\;2\;n\right)$	O $\left(4n^{5}\;\log\;2\;n\right)$	O $\left(4n^{5}\;\log\;3\;n\right)$	O $\left(4n^{5}\;\log\;5\;n\right)$	O $\left(4n^{5}\;\log\;7\;n\right)$	O $\left(4n^{5}\;\log\;9\;n\right)$
	BDLC	LLE	O $\left(2n^{5}\right)$	O $\left(2n^{5}\;\log\;n\right)$	O $\left(2n^{6}\;\log\;n\right)$	O $\left(2n^{6}\;\log\;2\;n\right)$	O $\left(2n^{6}\;\log\;4\;n\right)$	O $\left(2n^{6}\;\log\;6\;n\right)$	O $\left(2n^{6}\;\log\;8\;n\right)$
		DM	O $\left({4n}^{4}\right)$	O $\left({4n}^{4}\;\log\;n\right)$	O $\left({4n}^{5}\;\log\;n\right)$	O $\left({4n}^{5}\;\log\;2\;n\right)$	O $\left({4n}^{5}\;\log\;4\;n\right)$	O $\left({4n}^{5}\;\log\;6\;n\right)$	O $\left({4n}^{5}\;\log\;8\;n\right)$
		LE	O $\left({4n}^{5}\right)$	O $\left({4n}^{5}\;\log\;n\right)$	O $\left({4n}^{6}\;\log\;n\right)$	O $\left({4n}^{6}\;\log\;2\;n\right)$	O $\left({4n}^{6}\;\log\;4\;n\right)$	O $\left({4n}^{6}\;\log\;6\;n\right)$	O $\left({4n}^{6}\;\log\;8\;n\right)$
	Firefly	LLE	O $\left({2n}^{6}\;\log\;n\right)$	O $\left({2n}^{6}\;\log\;2\;n\right)$	O $\left({2n}^{7}\;\log\;2\;n\right)$	O $\left({2n}^{7}\;\log\;3\;n\right)$	O $\left({2n}^{7}\;\log\;5\;n\right)$	O $\left({2n}^{7}\;\log\;7\;n\right)$	O $\left({2n}^{7}\;\log\;9\;n\right)$
		DM	O $\left(4n^{5}\;\log\;n\right)$	O $\left(4n^{5}\;\log\;2\;n\right)$	O $\left(4n^{6}\;\log\;2\;n\right)$	O $\left(4n^{6}\;\log\;3\;n\right)$	O $\left(4n^{6}\;\log\;5\;n\right)$	O $\left(4n^{6}\;\log\;7\;n\right)$	O $\left(4n^{6}\;\log\;9\;n\right)$
		LE	O $\left({4n}^{6}\;\log\;n\right)$	O $\left({4n}^{6}\;\log\;2\;n\right)$	O $\left({4n}^{7}\;\log\;2\;n\right)$	O $\left({4n}^{7}\;\log\;3\;n\right)$	O $\left({4n}^{7}\;\log\;5\;n\right)$	O $\left({4n}^{7}\;\log\;7\;n\right)$	O $\left({4n}^{7}\;\log\;9\;n\right)$
	NLR	LLE	O $\left({2n}^{5}\;\log\;n\right)$	O $\left({2n}^{5}\;\log\;2\;n\right)$	O $\left({2n}^{6}\;\log\;2\;n\right)$	O $\left({2n}^{6}\;\log\;3\;n\right)$	O $\left({2n}^{6}\;\log\;5\;n\right)$	O $\left({2n}^{6}\;\log\;7\;n\right)$	O $\left({2n}^{6}\;\log\;9\;n\right)$
		DM	O $\left(4n^{4}\;\log\;n\right)$	O $\left(4n^{4}\;\log\;2\;n\right)$	O $\left(4n^{5}\;\log\;2\;n\right)$	O $\left(4n^{5}\;\log\;3\;n\right)$	O $\left(4n^{5}\;\log\;5\;n\right)$	O $\left(4n^{5}\;\log\;7\;n\right)$	O $\left(4n^{5}\;\log\;9\;n\right)$
		LE	O $\left({4n}^{5}\;\log\;n\right)$	O $\left({4n}^{5}\;\log\;2\;n\right)$	O $\left({4n}^{6}\;\log\;2\;n\right)$	O $\left({4n}^{6}\;\log\;3\;n\right)$	O $\left({4n}^{6}\;\log\;5\;n\right)$	O $\left({4n}^{6}\;\log\;7\;n\right)$	O $\left({4n}^{6}\;\log\;9\;n\right)$
	LR	LLE	O $\left({2n}^{4}\right)$	O $\left({2n}^{4}\;\log\;n\right)$	O $\left({2n}^{5}\;\log\;n\right)$	O $\left({2n}^{5}\;\log\;2\;n\right)$	O $\left({2n}^{5}\;\log\;4\;n\right)$	O $\left({2n}^{5}\;\log\;6\;n\right)$	O $\left({2n}^{5}\;\log\;8\;n\right)$
		DM	O $\left({4n}^{3}\right)$	O $\left({4n}^{3}\;\log\;n\right)$	O $\left({4n}^{4}\;\log\;n\right)$	O $\left({4n}^{4}\;\log\;2\;n\right)$	O $\left({4n}^{4}\;\log\;4\;n\right)$	O $\left({4n}^{4}\;\log\;6\;n\right)$	O $\left({4n}^{4}\;\log\;8\;n\right)$
		LE	O $\left({4n}^{4}\right)$	O $\left({4n}^{4}\;\log\;n\right)$	O $\left({4n}^{5}\;\log\;n\right)$	O $\left({4n}^{5}\;\log\;2\;n\right)$	O $\left({4n}^{5}\;\log\;4\;n\right)$	O $\left({4n}^{5}\;\log\;6\;n\right)$	O $\left({4n}^{5}\;\log\;8\;n\right)$
	Detrended FA	LLE	O$\left(n^{4}\right)$	O$\left(n^{4}\;\log\;n\right)$	O$\left(n^{5}\;\log\;n\right)$	O$\left(n^{5}\;\log\;2\;n\right)$	O$\left(n^{5}\;\log\;4\;n\right)$	O$\left(n^{5}\;\log\;6\;n\right)$	O$\left(n^{5}\;\log\;8\;n\right)$
		DM	O $\left({2n}^{3}\right)$	O $\left({2n}^{3}\;\log\;n\right)$	O $\left({2n}^{4}\;\log\;n\right)$	O $\left({2n}^{4}\;\log\;2\;n\right)$	O $\left({2n}^{4}\;\log\;4\;n\right)$	O $\left({2n}^{4}\;\log\;6\;n\right)$	O $\left({2n}^{4}\;\log\;8\;n\right)$
		LE	O $\left({2n}^{4}\right)$	O $\left({2n}^{4}\;\log\;n\right)$	O $\left({2n}^{5}\;\log\;n\right)$	O $\left({2n}^{5}\;\log\;2\;n\right)$	O $\left({2n}^{5}\;\log\;4\;n\right)$	O $\left({2n}^{5}\;\log\;6\;n\right)$	O $\left({2n}^{5}\;\log\;8\;n\right)$

Results show that our work approach can correctly detect the VT vs. NSR, PVC vs. NSR, and ST vs. NSR arrhythmia classes using the GMM with Adam hyperparameter tuning classifier with 98.38 %, 98.4 8 %, and 98.92 % accuracies. We evaluate the detection performance of our approach LLE, DM and LE with & without CS and HSO Feature Selection with GMM, EM, NLR, LR, BDLC, Detrended FA, Firefly and GSO and Adam hyperparameter tuning-based classifiers for cardiac arrhythmias detection with that of existing approaches in the references that are various dimensionality reduction techniques and classifiers using MIT-BIH Arrhythmia database, we identified eight existing ECG beat classification and detection approaches to compare with our work approach. Table 16 summarizes the number of classified cardiac arrhythmia types as well as the overall accuracies of our work approach and eight existing approaches.

**Table 16:** Summary of existing works for cardiac arrhythmias detection from MIT-BIH Database.

S.No	References	Features	Methodology (Classifiers)	Classes (Databases)	Overall Accuracy (%)
1	Martis et al. [12]	DWT + PCA + LDA	SVM with RBF	**5** - (Non-ectopic beat (N), Supra-ventricular ectopic beats (S), Ventricular ectopic beats (V), Fusion beat (F) and Unknown beat (Q))	96.92
2	Trans et al. [13]	Higher Order Statistics (HOS)	Fuzzy Hybrid Neural Network	**7 -** (Normal, Left bundle branch block beat, Right bundle branch block beat, Atrial premature beat, Premature ventricular contraction, Ventricular escape beat and Paced rhythm)	96.06
3	Sukanta et al. [14]	DWT	Neural Network	**4** - (Normal (N), left bundle branch block (LBBB), right bundle branch block (RBBB), Paced beats (P))	96.67
4	Rizal et al. [15]	Hjorth Descriptor (HD)	ANN and KNN	**3** - (Normal Sinus Rhythm (NSR), Atrial Fibrillation (AF) and Congestive Heart Failure (CHF))	93.3
5	Martis et al. [16]	Bispectrum and PCA	SVM with RBF	**5 -** (Normal, Right Bundle Branch Block (RBBB), Left Bundle Branch Block (LBBB), Atrial Premature Contraction (APC) and Ventricular Premature Contraction (VPC)).	93.48
6	Martis et al. [17]	Higher Order Cumulant (HOC) + PCA	Neural Network	**5** - (Normal (N), Right Bundle Branch Block (RBBB), Left Bundle Branch Block (LBBB), Atrial Premature Contraction (APC) and Ventricular Premature Contraction (VPC)).	94.52
7	Nazmy et al. [18]	ICA + Power Spectrum	FFNN, FIS and ANFIS	**6** - (Normal Sinus Rhythm (NSR), Premature Ventricular Contraction (PVC), Atrial Premature Contraction (APC), Ventricular Tachycardia(VT), Ventricular Fibrillation (VF) and Supraventricular Tachycardia (SVT)).	97.1
8	Dingfei et al. [19]	Auto Regressive Modeling	GLM	**6** - (Atrial premature Contraction (APC), Premature Ventricular Contraction (PVC), Supraventricular Tachycardia (SVT), Ventricular Tachycardia (VT) and Ventricular Fibrillation (VF) and Normal).	93.2
9	**In this paper**	LLE with CS feature selection	Firefly with GSO hyperparameter tuning	**4 -** (VT, PVC, ST change and NSR)	86.47 (VT vs. NSR)
					86.35 (PVC vs. NSR)
					87.12 (ST vs. NSR)
			Firefly with Adam hyperparameter tuning		91.71 (VT vs. NSR)
					91.59 (PVC vs. NSR)
					92.25 (ST vs. NSR)
10		LLE with HSO feature selection	Firefly with GSO hyperparameter tuning		93.75 (VT vs. NSR)
					93.75 (PVC vs. NSR)
					93.38 (ST vs. NSR)
			**Firefly with Adam hyperparameter tuning**		**98.38 (VT vs. NSR)**
					**98.48 (PVC vs. NSR)**
					**98.92 (ST vs. NSR)**

## 9 Conclusion

Cardiac Vascular Arrhythmias (VT, PVC and ST) are irregular cardiac rhythms. These types of cardiac arrhythmias are very dangerous to human health. Cardiac arrest, Chest pain, Fluttering, and Myocardial infarction are all symptoms of cardiac vascular diseases. In this work, ECG signals obtained from the MIT-BIH database are analyzed for Detection of VT, PVC, ST and Normal using different classifiers. The results show that the performance of the classifiers with hyperparameter tuning approaches is better than with and without CS and HSO feature selection. The higher accuracy of 98.38 % is achieved for the LLE dimensionality reduction with HSO feature selection in the GMM classifier with Adam hyperparameter tuning, as in the case of VT vs. NSR. The Adam hyperparameter tuning-based GMM classifier with LLE dimensionality reduction with HSO feature selection is maintained at 98.48 % accuracy as in detection for PVC vs. NSR. In the case of ST vs. NSR detection, an accuracy of 98.92 % is exhibited by the GMM classifier with Adam hyperparameter tuning with LLE dimensionality reduction and HSO feature selection. Adam's hyperparameter tuning-based GMM Classifier, which has 98.92 % accuracy in detecting ST vs. NSR cardiac disease, outperforms all other classifiers. Deep learning methods and the Convolution Neural Network (CNN) will be the future endeavours of this work.

## Funding

This research was conducted independently without financial support from any funding agencies.

## Data availability statement

The datasets generated and/or analyzed during the current study are available in the Physionet repository, https://archive.physionet.org/cgi-bin/atm/ATM.

## CRediT authorship contribution statement

**Gowri Shankar Manivannan:** Writing – review & editing, Writing – original draft, Visualization, Validation, Software, Resources, Project administration, Methodology, Investigation, Funding acquisition, Formal analysis, Data curation, Conceptualization. **Harikumar Rajaguru:** Supervision. **Rajanna S:** Supervision. **Satish V. Talawar:** Supervision.

## Declaration of competing interest

The authors declare that they have no known competing financial interests or personal relationships that could have appeared to influence the work reported in this paper.

## Figures and Tables

**Table:** 

Initialization: GSO maxiter, firefly maxiter, target, n, ${rand}_{\mathrm{int}}$, γ, $\alpha_{\min}$ and $\alpha_{0}$. For mk = 1: GSO maxiter Generate search space of GSO Estimate fitness level of system For m = 1: Firefly maxiter Tune the step factor (γ) using equation (66) Tune the addictive coefficient ($\alpha_{\min}$) using equation (66) Again generate search space of GSO Again estimate fitness level of system End for Generate confusion matrix Calculate error rate level Again using GSO, find appropriate hyperparameters scales End for

**Table:** 

Initialization: Adam maxiter, GMM maxiter, target, l, $R_{1}$, $R_{2}$, ∈, $\beta_{k}$, $\mu_{k}$, and $\sum_{k}$ For pq = 1: Adam maxiter Estimate fitness level of system Compute the appropriate mixture model weight For p = 1: GMM maxiter Tune the parameters using equations (22), (23), (24), (25), (26), (27), (28), (29), (30), (31) Again estimate fitness level of system End for Generate confusion matrix Calculate error rate level Again using Adam, find appropriate hyperparameters ranges using equations (68) to (74) End for

## Appendix

Algorithm 1

Algorithm 2
